# Genome-Wide Analysis and Identification of UDP Glycosyltransferases Responsive to Chinese Wheat Mosaic Virus Resistance in *Nicotiana benthamiana*

**DOI:** 10.3390/v16040489

**Published:** 2024-03-22

**Authors:** Xia Wang, Jin Yang, Haichao Hu, Tangyu Yuan, Yingjie Zhao, Ying Liu, Wei Li, Jiaqian Liu

**Affiliations:** 1 College of Plant Protection, Hunan Agricultural University, Changsha 410128, China; z0221wangxia@163.com (X.W.); huhdfs@163.com (H.H.); 2 State Key Laboratory for Quality and Safety of Agro-Products, Institute of Plant Virology, Ningbo University, Ningbo 315211, China; yjsamily@163.com (J.Y.); a15237558113@163.com (Y.Z.); ly1410147326@163.com (Y.L.); 3 Yantai Academy of Agricultural Science, No. 26 Gangcheng West Street, Fushan District, Yantai City 265500, China; ytyuantangyu@163.com

**Keywords:** UDP-glycosyltransferases (UGTs), *Nicotiana benthamiana* (*N. benthamiana*), expression pattern, Chinese wheat mosaic virus (CWMV)

## Abstract

Glycosylation, a dynamic modification prevalent in viruses and higher eukaryotes, is principally regulated by uridine diphosphate (UDP)-glycosyltransferases (UGTs) in plants. Although UGTs are involved in plant defense responses, their responses to most pathogens, especially plant viruses, remain unclear. Here, we aimed to identify UGTs in the whole genome of *Nicotiana benthamiana* (*N. benthamiana*) and to analyze their function in Chinese wheat mosaic virus (CWMV) infection. A total of 147 *NbUGTs* were identified in *N. benthamiana*. To conduct a phylogenetic analysis, the UGT protein sequences of* N. benthamiana *and *Arabidopsis thaliana* were aligned. The gene structure and conserved motifs of the UGTs were also analyzed. Additionally, the physicochemical properties and predictable subcellular localization were examined in detail. Analysis of cis-acting elements in the putative promoter revealed that *NbUGTs* were involved in temperature, defense, and hormone responses. The expression levels of 20 *NbUGTs* containing defense-related cis-acting elements were assessed in CWMV-infected *N. benthamiana*, revealing a significant upregulation of 8 *NbUGTs*. Subcellular localization analysis of three NbUGTs (NbUGT12, NbUGT16 and NbUGT17) revealed their predominant localization in the cytoplasm of *N. benthamiana* leaves, and NbUGT12 was also distributed in the chloroplasts. CWMV infection did not alter the subcellular localization of NbUGT12, NbUGT16, and NbUGT17. Transient overexpression of *NbUGT12*, *NbUGT16*, and *NbUGT17* enhanced CWMV infection, whereas the knockdown of *NbUGT12*, *NbUGT16* and *NbUGT17* inhibited CWMV infection in *N. benthamiana*. These *NbUGTs* could serve as potential susceptibility genes to facilitate CWMV infection. Overall, the findings throw light on the evolution and function of *NbUGTs*.

## 1. Introduction

Plants produce several secondary metabolites that are critical for their interactions with the environment, reproductive strategies, and defense responses [1]. Glycosylation, hydroxylation, acylation, and methylation play important roles in the diversity and complexity of plant secondary metabolites [2]. Glycosylation, which is essential for maintaining cellular homeostasis by regulating the levels, activities, and locations of key cellular metabolites [3], is widespread from viruses to higher eukaryotes [4,5]. During glycosylation, the glycosyl group from the donor molecule is transferred to the recipient molecule by glycosyltransferases (GTs) to form a glucoside bond, which further becomes converted into more stable and inactive storage forms [6,7]. Glycosylation produces oligosaccharides, polysaccharides, glycoproteins and glycolipids, and other glycoside compounds [8,9]. The process involves various types of glycosyl donors, including nucleotide-activated sugars such as UDP-glucose, UDP-galactose, UDP-rhamnose, UDP-xylose, and UDP-glucuronic acid. Glycosyl receptors can be carbohydrates or non-saccharide compounds, such as proteins, antibiotics, and phytohormones [10,11]. Glycosylation can affect the homeostasis of these compounds by modifying their chemical activity, degradation, or localization [10,12]. According to the recently updated CAZy (CAZy, http://www.cazy.org, accessed on 3 December 2023), GTs from different species have been classified into 117 enzyme families based on their amino acid sequence similarity, catalytic mechanism, substrate specificity, and the presence of conserved sequence motifs. Among them, UGTs belong to the largest GT family associated with secondary metabolites, such as phytohormones, terpenoids, and sterols [9,13,14]. UGTs are abundant in the plant kingdom, they are a highly differentiated and multi-lineated multi-gene family that is widely involved in the glycosylation of plant secondary metabolites [1]. UGTs are responsible for the glycosylation of small-molecule compounds and participate in multiple plant growth and development processes as well as plant defense responses [15].

With the development of sequencing technologies, an increasing number of UGT genes have been identified at the whole genome in various plant species, including *Arabidopsis thaliana*, *Brachypodium distachyon*, *Brassica rapa*, *Brassica oleracea*, rice, wheat, maize, soybean, cotton, alfalfa, and peach. The C-terminus of UGTs contains a highly conserved plant secondary product, the glycosyltransferase box (PSPG), composed of 44 amino acids [16]. The entire fold and core regions of plant UGTs are suggested to be conserved, because the C-terminal domain of UGTs mainly recognizes the same or similar glycosyl donors, while the N-terminal domain mainly recognizes specific receptor substrates [17,18]. Hence, the PSPG motif plays a critical role in regulating the glycosylation of specific phytohormones, defense compounds, and other secondary metabolites in plants [19,20]. Phytohormones are the key endogenous factors mediating the plant stress response, which is the integration center of plants to cope with environmental stimuli and plays an important role in plant defense responses [15]. UGTs indirectly regulate biotic stress responses by glycosylating phytohormones, including jasmonic acid (JA) and salicylic acid (SA), or phytohormone-related compounds. For example, the overexpression of *UGT74F2* in *Arabidopsis thaliana* reduced free SA levels and resistance to *Pseudomonas syringae* (*P. syringae*) infection; conversely, the knockout of *UGT74F2* had the opposite effect [21,22]. The knockout of *UGT74J1* in *Oryza sativa* resulted in increased SA levels and promoted resistance to *Magnaporthe oryzae* [23]. The disruption of the expression of methyl salicylate (MeSA)-specific *UGT71C3* in *Arabidopsis thaliana* enhanced systemic acquired resistance to *P. syringae* by increasing MeSA and free SA levels [24]. UGTs with secondary metabolite activities are considered important for plant resistance to pathogens [15]. For instance, *Arabidopsis thaliana UGT71C1* can glycosylate the secondary metabolite flavonoids, thus affecting the level of reactive oxygen species (ROS) and reducing the damage to plants caused by excessive ROS during pathogen invasion [25]. Silencing of *Twi1*, a homolog of TOGT in *Nicotiana tabacum (N. tabacum)*, resulted in an increase in total scopoletin and decreased resistance to tomato spotted wilt virus in *Solanum lycopersicum* [26].

CWMV was discovered and identified at the end of the last century on winter wheat in Shandong Province, China [27,28,29]. It naturally infects wheat plants, leading to typical mosaic symptoms, and is one of the most common pathogens causing wheat yellow mosaic disease in China. CWMV is a member of the genus *Furovirus* and is spread by the plasmodiophorid *Polymyxa graminis (P. graminis*) [27,30]. According to the life history of *P. graminis* [31], its resting sporangium can survive in the residue or soil after wheat harvest. Under suitable environmental conditions, these spores can germinate and produce zoospores that colonize wheat roots. The CWMV particles carried by *P. graminis* can infect the roots during this stage, leading to proliferation and systemic infection. CWMV is comprised of two positive-sense single-stranded RNAs, RNA1 and RNA2. RNA1 possesses 7147 nucleotides and includes three major predicted open reading frames that encode the replication-associated protein, RNA-dependent RNA polymerase (RdRp), and a movement protein (MP), which are required for viral replication and movement. RNA2 contains 3563–3569 nucleotides according to different isolates and encodes four proteins, coat protein (CP), CP–read-through (CP-RT), N-terminal extension CP (N-CP), and cysteine-rich protein (CRP) [30]. CWMV and wheat yellow mosaic virus (WYMV) often co-infect wheat, posing a serious threat to grain production safety [29]. In laboratory experiments, it has been demonstrated that CWMV can infect *N. benthamiana* through mechanical inoculation and cause symptoms similar to those of CWMV-infected wheat, making it a common model system for investigating the interaction between CWMV and plants [32].

Some UGTs are involved in the regulation of host resistance to viruses in plants, such as the overexpression of *Togt1* and *Togt2* in *N. tabacum*, which increased resistance to tobacco mosaic virus and potato virus Y [33,34]. However, only a few UGT genes have been reported to regulate viral disease resistance in wheat. In this study, UGTs from *N. benthamiana* were identified at the whole-genome level. Subsequently, phylogenetic and gene structure analyses were conducted, followed by the prediction of subcellular localization and the analysis of cis-acting elements in the gene promoter. The expression levels of some UGTs were measured in the CWMV infection time cross, and UGTs responding to CWMV infection in *N. benthamiana* were screened. Finally, the regulatory effects of three UGTs on CWMV were investigated. This study provides a reference for the role of UGTs in regulating viral disease resistance in plants.

## 2. Materials and Method

### 2.1. Identification and Bioinformatics Analysis of NbUGTs

Protein sequences of 19 *Arabidopsis thaliana* UGT family members were downloaded from The Arabidopsis Information Resource (https://www.arabidopsis.org/, accessed on 10 September 2022) and used as templates to perform a BLASTP search for all UGTs in *N. benthamiana*. The possible genome of *N. benthamiana* was identified using the hidden Markov model (HMM) profile of the UDPGT superfamily domain (PF00201), which was downloaded from the Sol Genomics Network (https://solgenomics.net/, accessed on 12 September 2022). The PSPG domain was used as a further screening criterion, and after ruling out 65 genes, 147 *NbUGTs* were identified. Then, phylogenetic analysis was performed based on the full-length protein sequences of 19 *Arabidopsis thaliana* UGT family members and 147 *NbUGTs* using MEGA11.0 software [8,12] with the neighbor-joining (NJ) method, and a bootstrap test was carried out with 1000 iterations [35]. In addition, the coding sequence (CDS) lengths, isoelectric points (pIs), and molecular weights (MWs) of the NbUGTs were predicted using ExPASy [36], and the subcellular localizations of the NbUGTs were predicted using the tool in the website (http://cello.life.nctu.edu.tw/cello.html, accessed on 16 September 2022).

### 2.2. Analysis of Conserved Motifs, Gene Structure, and Conserved Domains of NbUGTs

The conserved motifs of the NbUGTs were analyzed using the MEME program, with the maximum number of motifs set to 10 [37] (Table S1). The gene structures of the *NbUGTs* were analyzed and visualized using TBtools-II v1.108 with the *N. benthamiana* genome annotation file. The conserved domains of the genes were analyzed using the TBtools-II v1.108.

### 2.3. Prediction of Cis-Acting Elements in the Putative Promoter Regions

To investigate cis-acting elements in the putative promoter regions of the obtained *NbUGTs*, 2 kb genomic DNA sequences upstream of the initiation codon (ATG) of all *NbUGTs* were downloaded from the Sol Genomics Network (https://solgenomics.net/, accessed on 18 September 2022). Cis-regulatory elements in the putative promoter sequences were analyzed using PlantCARE (http://bioinformatics.psb.ugent.be/webtools/plantcare/html/, accessed on 18 September 2022) [38] and visualized by TBtools-II v1.108.

### 2.4. Plant Culture and Virus Inoculation

*N*. *benthamiana* needed to be cultured in a growth chamber at 15 ± 2 °C under a 16 h/8 h light/dark photoperiod when inoculated with CWMV, while the other plants were grown in soil inside a growth chamber maintained at 25 ± 2 °C with a 16 h/8 h light/dark cycle and 65 ± 5% relative humidity. To inoculate CWMV, briefly, plasmids pCB-35S-R1 and pCB-35S-R2, respectively, containing CWMV RNA1 and RNA2 full-length sequences were individually transformed into the *Agrobacterium tumefaciens* strain GV3101. The Agrobacterium cultures were grown individually overnight at 28 °C. The resulting Agrobacterium cultures were pelleted and resuspended in an infiltration buffer (10 mM MES, pH of 5.7, 10 mM MgCl_2_, 0.2 mM acetosyringone) at room temperature (OD_600_ = 0.6–0.8). Agrobacterium harboring pCB-35S-R1 was mixed with Agrobacterium harboring pCB-35S-R2 in a 1:1 ratio. Mixed cultures were individually infiltrated into *N. benthamiana* leaves. A TRV-based VIGS system in *N. benthamiana* was used in this study, while the plasmids pTRV1 and pTRV2 or pTRV2-*NbUGT12*, *NbUGT16*, and *NbUGT17* were individually transformed into the *Agrobacterium tumefaciens* strain GV3101. The Agrobacterium cultures were grown individually overnight at 28 °C. Then, the cultures were pelleted and resuspended in the infiltration buffer at room temperature (OD_600_ = 0.3–0.6). Agrobacterium harboring pTRV1 was mixed with Agrobacterium harboring pTRV2 or pTRV2-NbUGT12, pTRV2-NbUGT16, and pTRV2-NbUGT17 in a 1:1 ratio. Mixed cultures were infiltrated into *N. benthamiana* leaves. Moreover, H2B-RFP transgenic *N. benthamiana* plants were also used in this study. These plants can express a nuclear marker H2B-RFP, and are always used for subcellular localization research [39,40].

### 2.5. Plasmid Construction

Firstly, *NbUGT12*, *NbUGT16*, and *NbUGT17* were cloned from the cDNA of *N. benthamiana*. The cloned fragments were recombined into the Ti vector plasmid according to the instructions of the pEASY^®^-Blunt Zero Cloning Kit (TransGen Biotech, Beijing, China). Then, the Ti-NbUGT12, Ti-NbUGT16, and Ti-NbUGT17 plasmids were used as templates to amplify the full-length and silent fragments of the genes, respectively. Next, the *NbUGT12*, *NbUGT16*, and *NbUGT17* CDS full-length fragments were sequentially inserted into the intermediate vector Donor 207 and the GFP-tagged PGWB505 vector according to the Gateway series construction method. Finally, the recombinant NbUGT12-GFP, NbUGT16-GFP, and NbUGT17-GFP plasmids were transferred into the Agrobacterium by electroporation, and the corresponding Agrobacterium with NbUGT12-GFP, NbUGT16-GFP, and NbUGT17-GFP plasmids were obtained. To silence *NbUGT12*, *NbUGT16*, and *NbUGT17* in *N. benthamiana*, approximately 300 bp fragments of *NbUGT12*, *NbUGT16*, and *NbUGT17* were inserted into the pTRV2 vector, respectively. The silenced fragments were designed on the Sol Genomics Network (https://solgenomics.net/, accessed on 15 October 2022) and their sequences are listed in Table S2. Two endonuclease BamHI and SmaI sequences were added when designing the primers for amplifying the fragments. Then, the amplified fragments and the pTRV2 vector plasmid were all digested with the two enzymes and recovered. Then, the pTRV2-NbUGT12, pTRV2-NbUGT16, and pTRV2-NbUGT17 fragments were ligated to the digested pTRV2 plasmid using T4 DNA Ligase (Thermo Scientific, Waltham, MA, USA), respectively. Finally, the recombinant plasmids were transferred into Agrobacterium.

### 2.6. RNA Extraction and Quantitative Reverse-Transcription PCR (qRT-PCR) Assay

The total RNA of the *N. benthamiana* tissue samples was extracted using the HiPure Plant RNA Mini Kit (Magen, Guangzhou, China). First-strand cDNA was synthesized using the First-Strand cDNA Synthesis Kit (Vazyme, Nanjing, China). The qRT-PCR was carried out using Hieff^®^ qPCR SYBR Green Master Mix (Yeasen, Shanghai, China) on an Applied Biosystems Quantstudio 6 Flex system (Applied Biosystems, Foster City, CA, USA), and the relative expression levels of the assayed genes were calculated using the 2^−ΔΔCt^ method. Each treatment had three biological replicates and three technical replicates. The primer sequences used in this study are listed in Table S3.

### 2.7. Western Blot (WB) Assay

The total protein was extracted from *N. benthamiana* leaves and homogenized in a lysis buffer containing 2% β-mercaptoethanol, 6% sodium dodecyl sulfate (SDS), and 100 mM Tris-HCl (pH of 8.8). The protein samples were individually mixed with SDS loading buffer and boiled for approximately 8 min. Protein samples were separated by SDS–polyacrylamide gel electrophoresis (PAGE) and transferred to nitrocellulose membranes. The blots were incubated in a blocking buffer (5% skim milk in 1×PBS) for 1 h, followed by detection using specific anti-GFP (TransGen Biotech, Beijing, China) or anti-CWMV CP primary antibody and then an HRP-conjugated anti-mouse or anti-rabbit secondary antibody (Abbkine Scientific, California, USA). The detection signal was visualized using an Amersham Imager 680 machine (GE Healthcare BioSciences, Pittsburgh, PA, USA). In addition, the specific primary antibody for detecting CWMV CP was prepared and preserved in our laboratory.

### 2.8. Confocal Fluorescence Microscope Observation

The confocal fluorescence microscope (TCS SP8 X) was used to observe the expression of fluorescently labeled genes, and the LAS X application was used for photographing and analysis. Briefly, the inoculation leaves of *N. benthamiana* were sampled with a puncher; then, the samples were placed on a glass slide and covered with a coverslip before being observed under the microscope. LAS X is equipped with channels to observe different fluorescence, such as GFP, RFP, and YFP, and it also has a special chloroplast channel that can be used to observe whether the gene can express in the chloroplasts [41]. When detecting the fluorescence signal, the excitation and emission wavelengths of the GFP fluorescence were 488 nm and 500–555 nm, respectively, the RFP fluorescence excitation and emission wavelengths were 552 nm and 565–620 nm, respectively, while those of the chloroplast fluorescence excitation and emission wavelengths were 552 nm and 670–730 nm, respectively.

### 2.9. Chloroplast Extraction

The chloroplasts were extracted using the MinuteTM Chloroplast Isolation Kit (Invent Biotechnologies, Beijing, China) according to the manufacturer’s instructions. Subsequently, the resulting samples were analyzed using WB analysis [41].

## 3. Results

### 3.1. Identification and Phylogenetic Analysis of NbUGTs

The genome sequences of *N. benthamiana* were downloaded from the Sol Genomics Network (https://solgenomics.net/, accessed on 12 September 2022). Initially, 212 possible *NbUGTs* were identified using the HMM profile of the UDPGT superfamily domain (PF00201) in *N. benthamiana*. After removing 19 sequences that lacked the PSPG domain, 193 *NbUGTs* were obtained. Furthermore, truncated UGTs and genes with less than 50% identity with the 44 amino acids of the PSPG motif were removed. In total, 147 UGT genes were identified in *N. benthamiana*.

**Figure 1:**
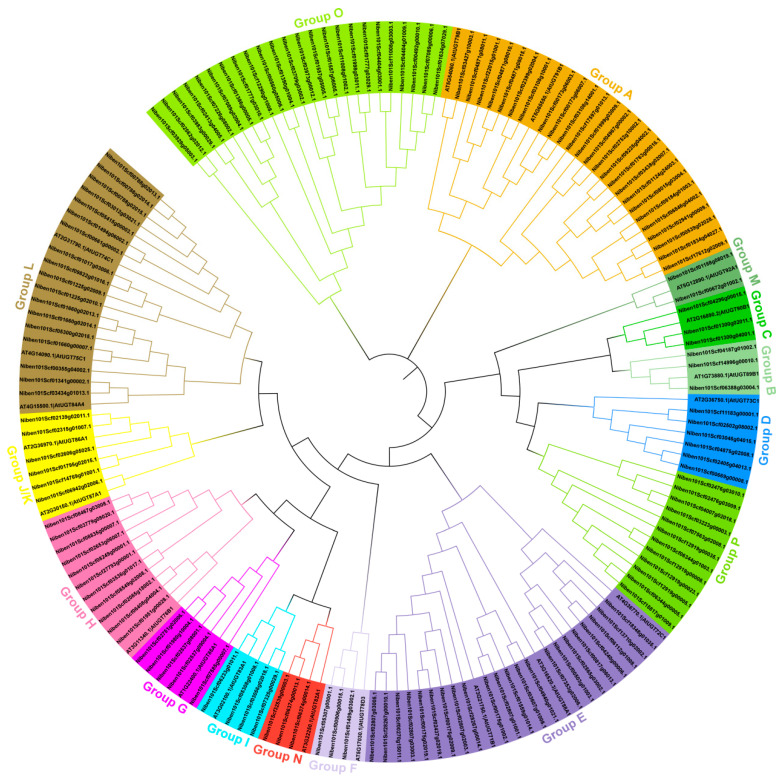
Phylogenetic analysis of NbUGT gene family. The MUSCLE and MEGA11.0 software were used for the sequence alignment and construction of the phylogenetic tree using the full-length sequences of 147 NbUGTs and 19 *Arabidopsis thaliana* UGT genes.

To determine the evolutionary relationships between the UGT family genes in *N. benthamiana* and *Arabidopsis thaliana*, a phylogenetic tree was constructed based on the amino acid sequence similarity of 19 UGTs in *Arabidopsis thaliana* that were distributed in 14 different groups from A–N and 147 NbUGTs using MEGA11.0 [8,12]. The results showed that the 147 NbUGTs were classified into 16 major phylogenetic groups, while all 14 conserved evolutionary groups (A–N) originally described in *Arabidopsis thaliana* were included in *N. benthamiana* (Figure 1). The number of NbUGTs in each group varied, with the largest one, Group A, containing 25 NbUGTs, and the smallest one, Group M, having only 2 members (Figure 1). In addition, two new evolutionary groups not found in *Arabidopsis thaliana* were identified in *N. benthamiana*: Group O with 24 members and Group P with 12 members (Figure 1).

### 3.2. Prediction of the Subcellular Localization and Physicochemical Properties of NbUGTs

The *NbUGTs*’ physicochemical properties, including the MW, protein length (PL), pI, CDS length, and intron number (IN), were determined using ExPASy (Table 1). The MW of these NbUGTs varied from 30.55219 kDa (*Niben101Scf04967g00002.1*) to 91.45533 kDa (*Niben101Scf03046g04015.1*), with a range of 270 to 801 amino acids (aa), respectively. The CDS lengths of the *NbUGTs* ranged from 813 bp (*Niben101Scf04967g00002.1*) to 2406 bp (*Niben101Scf03046g04015.1*). A significant number (132) of the NbUGTs were acidic proteins (pI < 7). Out of these NbUGTs, 81.63% (120) either lacked or contained only one intron. The subcellular localizations predicted on the website (http://cello.life.nctu.edu.tw/cello.html, accessed on 16 September 2022) are shown in Table 1. The results showed that the NbUGTs were most likely localized in the cytoplasm, followed by the plasma membrane, chloroplasts, nucleus, and outer membrane (Table 1).

### 3.3. Gene Structure and Conserved Motifs Analysis of NbUGTs

**Figure 2:**
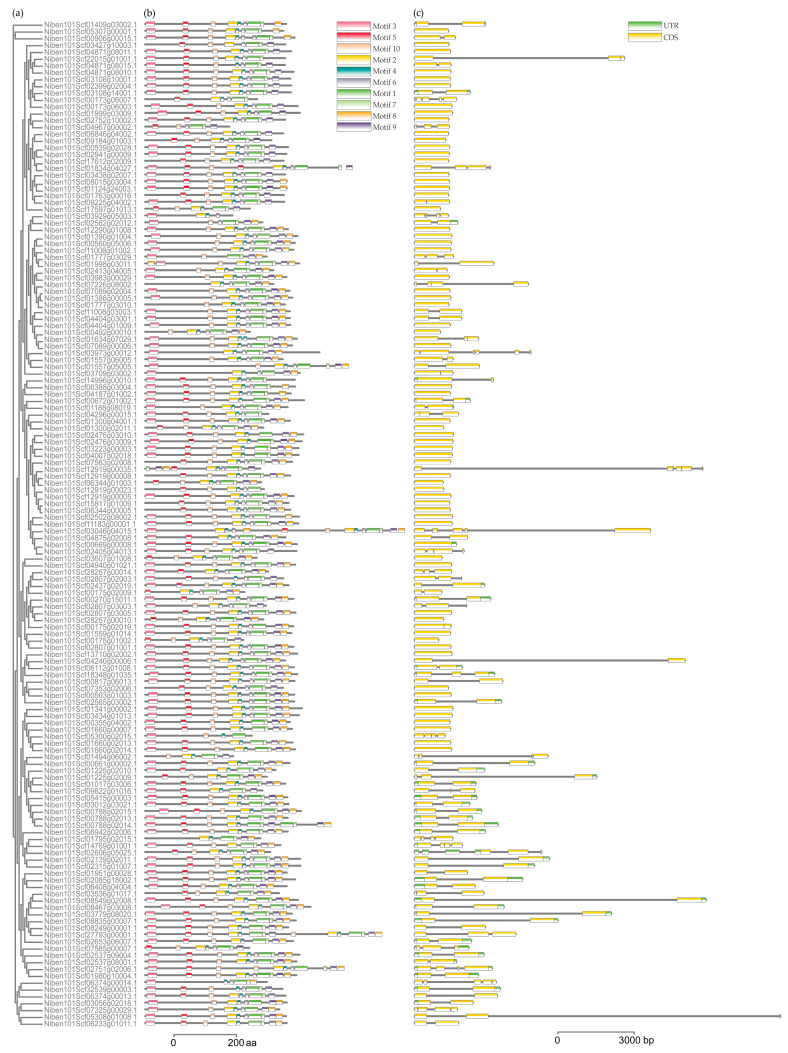
Analysis of conserved motifs and gene structure of NbUGTs. (**a**) Phylogenetic tree constructed using the NbUGT protein sequences. (**b**) Ten types of conserved motifs were predicted in the NbUGT protein sequences. Different motifs are shown in different color boxes. The sequence information for each motif is provided in Supplementary Table S1. (**c**) The gene structure of *NbUGTs* (untranslated regions, exons, and introns are shown as light green boxes, yellow boxes, and horizontal lines, respectively).

Ten conserved motifs were identified among the 147 NbUGTs using online MEME analysis. The majority of members contained five to nine motifs and always started with motif three and ended with motif two. Only a few genes were different, for example, *Niben101Scf03929g05003.1*, which contained only four motifs (Figure 2a,b). Additionally, most of the 147 members did not have any introns or contained only one intron (Figure 2c).

### 3.4. Identification of Conserved Domains and Cis-Acting Elements in the Putative Promoter of NbUGTs

**Figure 3:**
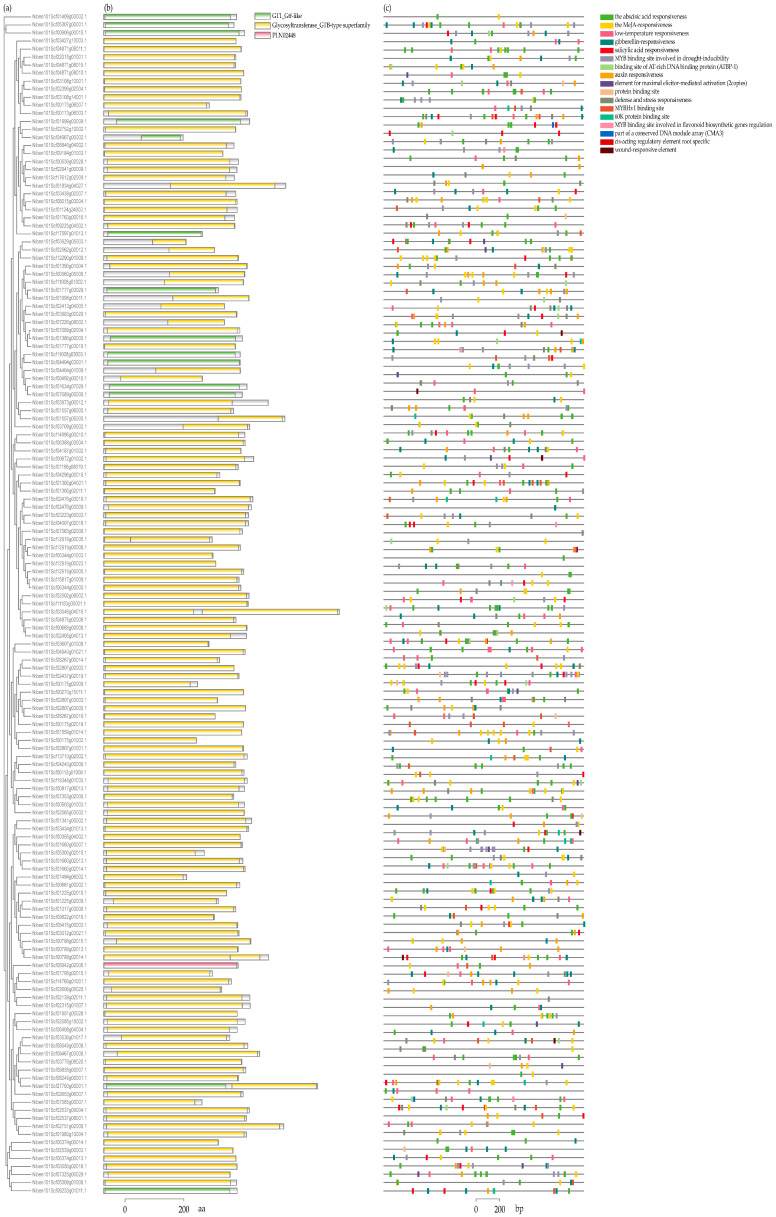
Predicted conserved domains and cis-acting elements of NbUGTs. (**a**) Phylogenetic tree constructed using the NbUGT protein sequences. (**b**) The conserved domains of the NbUGTs were analyzed through TBtools-II v1.108 software. (**c**) The type, quantity, and position of predicted cis-acting elements in the putative promoter regions of NbUGTs. Different elements are shown in different color boxes.

Among the 147 *NbUGTs*, 90.48% (133) contained the Glycosyltransferase_GTB-type superfamily domain, 9.52% (14) had a GT1_Gtf-like domain, and only 1 *NbUGT* (*Niben101Scf06942g02006.1*) contained the PLN02448 domain. This indicated a high level of conservation among the NbUGTs (Figure 3a,b). To investigate the possible regulatory mechanisms of the NbUGTs, the PlantCARE web server was used to search for possible cis-acting elements with the 2000 bp putative promoter regions of the NbUGTs. A total of 17 types of cis-acting elements were identified. These elements were associated with environmental stress, hormonal responses, development, and defense responses (Figure 3c).

### 3.5. Expression Patterns of NbUGTs during CWMV Infection

**Figure 4:**
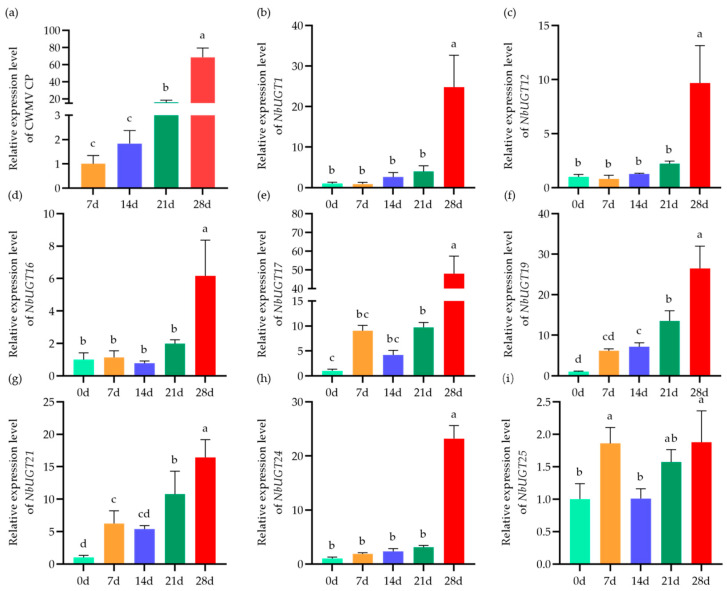
The expression patterns of 8 *NbUGTs* in CWMV-infected *N. benthamiana* plants. (**a**) The accumulation of CWMV CP in the assayed systemic leaves of *N. benthamiana* infected with CWMV at 7 to 28 days. (**b**–**i**) Relative expression levels of *NbUGT1*, *NbUGT12*, *NbUGT16*, *NbUGT17*, *NbUGT19*, *NbUGT21*, *NbUGT24*, and *NbUGT25* in CWMV-infected *N. benthamiana* plants at 0 to 28 dpi. Each treatment had three biological replicates; the data presented are the means ± SD, determined using Student’s *t*-test. Different letters show statistically significant differences (*p* < 0.05, Tukey’s test).

To further clarify the functions of the *NbUGTs* in response to CWMV infection, the expression patterns of the partial *NbUGTs* were analyzed following CWMV infection in *N. benthamiana*. The plants were inoculated with CWMV and cultured in an artificial climate incubator at 15 °C. Then, the total RNA was extracted from their systemic leaves on 0, 7, 14, 21, and 28 days post-infection (dpi), respectively, followed by qRT-PCR analysis of 20 *NbUGTs* containing defense-related cis-acting elements (the corresponding gene names are listed in Table S4). At 21 dpi, there was a significant increase in CWMV CP accumulation. After an additional 7 days, the accumulation level of CWMV CP increased by nearly 70 times compared to that on day 7 (Figure 4a). Through the qRT-PCR assay, 8 *NbUGTs* were identified as potential responders to CWMV infection (Figure 4), whereas the expression levels of the remaining 12 NbUGT genes did not show significant changes during CWMV infection (Figure S1). The expression patterns of *NbUGT1*, *NbUGT12*, *NbUGT16,* and *NbUGT24* were similar, displaying a minor, non-significant difference at 0–21 dpi but showing a significant increase at 28 dpi (Figure 4b–d,h). However, the expression profiles of *NbUGT17*, *NbUGT21,* and *NbUGT25* showed an initial increase, followed by a decrease, and then an increase again (Figure 4e,g,i). *NbUGT19* exhibited a significant increase in expression over time, reaching a level 26 times higher than that on day 0 at 28 dpi (Figure 4f). In summary, eight *NbUGTs* were significantly upregulated during CWMV infection, suggesting their potential involvement in the interaction between CWMV infection and the defense response of *N. benthamiana*. Subsequently, according to the expression patterns, the eight significantly upregulated genes were divided into the following three groups: the high upregulation, medium upregulation, and low upregulation categories containing three, three, and two genes, respectively. We then randomly selected one in each of the groups for functional studies. Consequently, *NbUGT12*, *NbUGT16*, and *NbUGT17* were picked for further investigation. In addition, it is worth noting that *NbUGT12*, *NbUGT16*, and *NbUGT17* all contain one intron (Figure S2), which is consistent with the predicted results (Table 1).

### 3.6. Subcellular Localization of NbUGT12, NbUGT16, and NbUGT17

**Figure 5:**
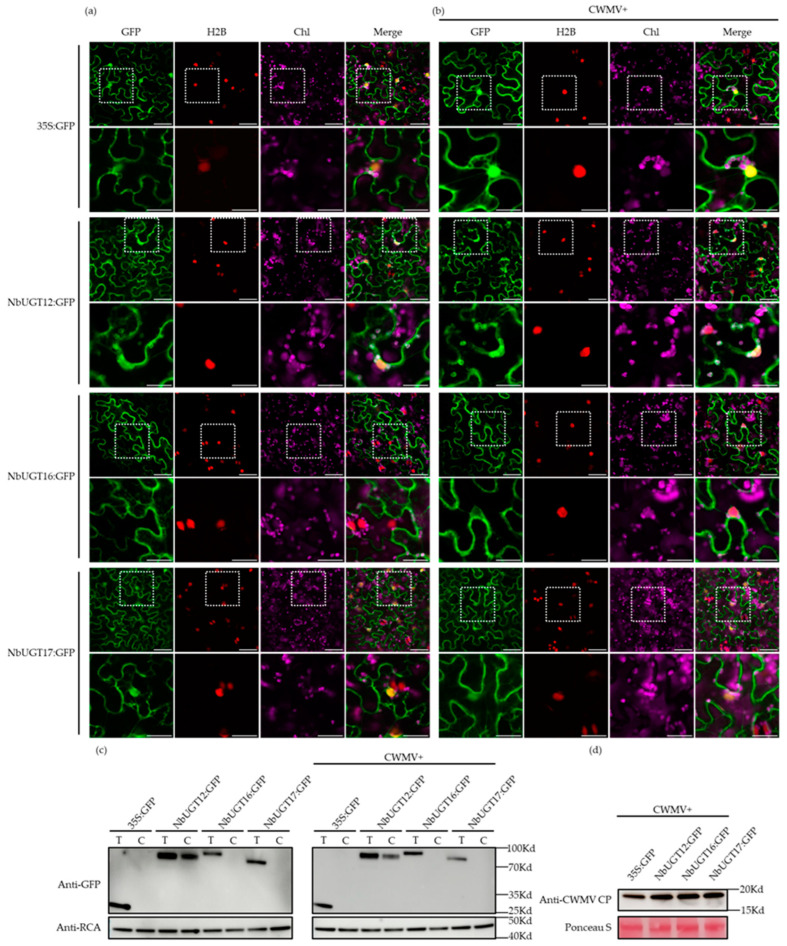
Analyzing the subcellular localization of NbUGT12, NbUGT16, and NbUGT17. (**a**) Subcellular localization of NbUGT12-GFP, NbUGT16-GFP, and NbUGT17-GFP in H2B-RFP transgenic *N. benthamiana* epidermal cells. Confocal images were taken at 72 hpi. Scale bar = 50 μm. The corresponding region in the white box was magnified below it. Scale bar = 25 μm, Chl (chloroplast). (**b**) Subcellular localization of NbUGT12-GFP, NbUGT16-GFP, and NbUGT17-GFP at 15 °C in H2B-RFP transgenic *N. benthamiana* leaves infected with CWMV at 7 dpi. Scale bar = 50 μm. The corresponding region in the white box was magnified below it. Scale bar = 25 μm. (**c**) The chloroplast proteins of *N. benthamiana*-inoculated leaves were analyzed by the WB assay and RCA and were used as a chloroplast maker. T (total protein); C (chloroplast). (**d**) The accumulation of CWMV CP in the assayed *N. benthamiana*-inoculated leaves was analyzed by the WB assay using CWMV CP-specific antibody. Ponceau S was used to visualize the sample loadings.

The protein functions are closely related to their subcellular localization. To analyze the characteristics and effects of the identified NbUGTs, their subcellular localizations were investigated using a website (http://cello.life.nctu.edu.tw/cello.html, accessed on 16 September 2022). Based on the predictions, NbUGT12 and NbUGT16 are most likely localized in the cytoplasm, while NbUGT17 has the potential localization in the cell membrane and cytoplasm (Table 1). Here, NbUGT12-GFP, NbUGT16-GFP, and NbUGT17-GFP were expressed in the transgenic *N. benthamiana* leaves (expressing H2B-RFP, a nuclear marker) via agroinfiltration. Non-fused GFP was used as a control. The inoculated leaves were collected at 72 h post-infiltration (hpi) to observe subcellular localization under a confocal microscope. The results revealed that NbUGT12-GFP was located in the cytoplasm and chloroplasts, NbUGT16-GFP was mainly located in the cytoplasm, whereas NbUGT17-GFP was present in the cytoplasm and nucleus (Figure 5a). To analyze the relationship between *NbUGTs* and CWMV, we tested whether CWMV affected the subcellular localization of the three proteins. Consequently, NbUGT12-GFP, NbUGT16-GFP, and NbUGT17-GFP were co-injected with CWMV in H2B-RFP transgenic *N. benthamiana* leaves using agroinfiltration. In brief, Agrobacterium individually harboring pCB-35S-R1 and pCB-35S-R2 were mixed with Agrobacterium harboring NbUGT12-GFP, NbUGT16-GFP, and NbUGT17-GFP in a 1:1:1 ratio, respectively. Mixed cultures were individually infiltrated into the plants leaves. At 7 dpi, the inoculated leaves were observed by confocal microscopy. The results showed no significant changes (Figure 5b). In addition, to determine whether these three proteins can express in chloroplasts, the chloroplast protein was extracted and analyzed using the WB assay. The results showed that NbUGT12 was indeed localized in the chloroplasts, while the control proteins, NbUGT16 and NbUGT17, did not express in the chloroplasts, regardless of CWMV infection (Figure 5c,d). In general, NbUGT12, NbUGT16, and NbUGT17 were primarily expressed in the cytoplasm, although NbUGT12 was also distributed in the chloroplasts and did not exhibit changes during CWMV infection.

### 3.7. NbUGT12, NbUGT16, and NbUGT17 Positively Regulate CWMV Infection in N. benthamiana

**Figure 6:**
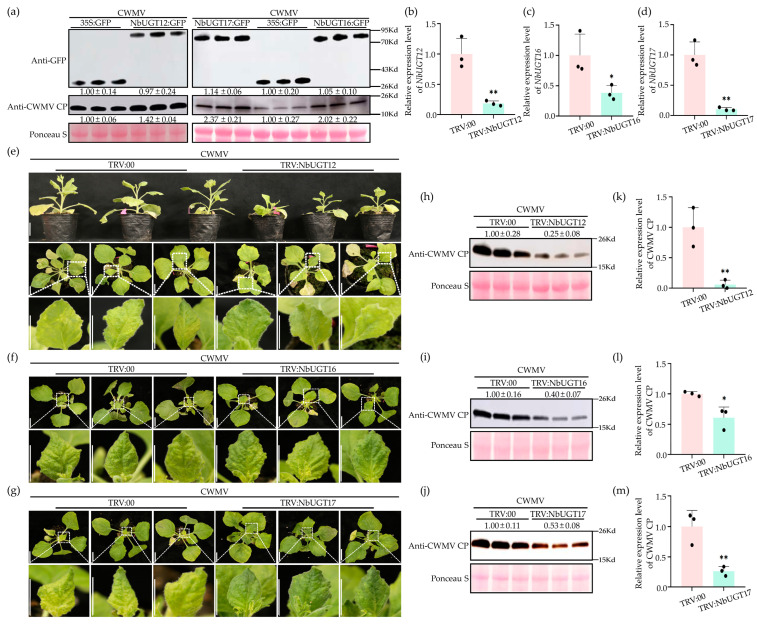
*NbUGT12*, *NbUGT16,* and *NbUGT17* positively regulate CWMV infection in *N. benthamiana*. (**a**) NbUGT12-GFP, NbUGT16-GFP, and NbUGT17-GFP were co-injected with CWMV in *N. benthamiana* leaves, respectively. Plants inoculated with GFP + CWMV were used as controls. The protein expression levels of NbUGT12-GFP, NbUGT16-GFP, and NbUGT17-GFP were detected by anti-GFP immunoblots. The accumulation levels of CWMV CP in the assayed plants were determined by WB analysis using a CWMV CP-specific antibody. Gray analysis was performed on the WB bands using ImageJ 1.8.0, each treatment had three biological replicates, and the data presented are the means ± SD. Ponceau S was used to visualize sample loadings. (**b**–**d**) qRT-PCR assay was used to determine the relative expression levels of *NbUGT12*, *NbUGT16*, and *NbUGT17* in TRV: NbUGT12-, TRV: NbUGT16-, TRV: NbUGT17-, and TRV: 00-inoculated *N. benthamiana* plants. (**e**–**g**) Phenotypes of *N. benthamiana* plants inoculated with TRV: NbUGT12+CWMV, TRV: NbUGT16+CWMV, and TRV: NbUGT17+CWMV at 30 dpi. Plants inoculated with TRV: 00+CWMV were used as controls. Scale bar = 2 cm. (**h**–**j**) The accumulation of CWMV CP in TRV: NbUGT12+CWMV-, TRV: NbUGT16+CWMV-, and TRV: NbUGT17+CWMV-inoculated assayed plants were analyzed by WB. Plants inoculated with TRV: 00+CWMV acted as controls. Gray analysis was performed on the WB bands using ImageJ 1.8.0, each treatment had three biological replicates, and the data presented are the means ± SD. Ponceau S was used to show the protein loadings. (**k**–**m**) Transcriptional levels of CWMV in TRV: NbUGT12+CWMV-, TRV: NbUGT16+CWMV-, and TRV: NbUGT17+CWMV-inoculated assayed plants were detected by qRT-PCR assay. Plants inoculated with TRV: 00+CWMV acted as controls. Each treatment had three biological replicates, and the data presented are the means ± SD, determined using Student’s *t*-test. * *p* < 0.05, ** *p* < 0.01.

To investigate the role of *NbUGT12*, *NbUGT16*, and *NbUGT17* in CWMV infection, NbUGT12-GFP, NbUGT16-GFP, and NbUGT17-GFP were co-injected with CWMV into *N. benthamiana*, respectively. Plants inoculated with GFP and CWMV were used as controls. After confirming the expression of these proteins using a confocal fluorescence microscope at 7 dpi, the total protein of the inoculated leaves was extracted. Then, the accumulation levels of NbUGT12-GFP, NbUGT16-GFP, and NbUGT17-GFP, and GFP and CWMV CP were detected by the WB assay. The results indicated that the accumulation levels of NbUGT12-GFP, NbUGT16-GFP, and NbUGT17-GFP were comparable to their respective controls, while the accumulation level of CWMV CP significantly increased in NbUGT12-GFP+CWMV-, NbUGT16-GFP+CWMV-, and NbUGT17-GFP+CWMV-inoculated plants compared to that in the control plants (Figure 6a).

*NbUGT12*, *NbUGT16*, and *NbUGT17* were also silenced in *N. benthamiana* by the TRV-mediated VIGS assay. Firstly, the infectious clones of TRV-NbUGT12, TRV-NbUGT16, and TRV-NbUGT17 were inoculated in *N. benthamiana*, respectively, with TRV: 00 serving as the control. The total RNA was extracted from the systemic leaves at 7 dpi, and qRT-PCR analysis was performed. The results showed that the expression levels of *NbUGT12*, *NbUGT16*, and *NbUGT17* were reduced by approximately 80%, 60%, and 90% in TRV: NbUGT12-, TRV: NbUGT16-, and TRV: NbUGT17-infected plants, respectively, compared to those in TRV: 00-infected plants (Figure 6b–d). Subsequently, the plants successfully silenced *NbUGT12*, *NbUGT16*, and *NbUGT17* were inoculated with CWMV, respectively. After another 30 dpi, the TRV: NbUGT12+CWMV-, TRV: NbUGT16+CWMV-, and TRV: NbUGT17+CWMV-inoculated plants exhibited milder CWMV disease symptoms than their controls (Figure 6e–g). In addition, TRV: NbUGT12+CWMV-inoculated plants were shorter than the controls, indicating that *NbUGT12* may also play a significant role in plant growth (Figure 6e). The total RNA and protein were extracted from the systemic leaves. Then, the accumulation levels of CWMV CP in these plants were analyzed by WB and qRT-PCR assays. The WB assay revealed a lower accumulation of CWMV CP in TRV: NbUGT12+CWMV-, TRV: NbUGT16+CWMV-, and TRV: NbUGT17+CWMV-inoculated plants than in TRV: 00+CWMV-inoculated plants (Figure 6h–j). Moreover, qRT-PCR analysis revealed that the transcriptional levels of CWMV CP were downregulated by approximately 95%, 40%, and 75% in the TRV: NbUGT12+CWMV-, TRV: NbUGT16+CWMV-, and TRV: NbUGT17+CWMV-inoculated plants, respectively, compared those in the control plants (Figure 6k–m). In summary, these findings suggested that *NbUGT12*, *NbUGT16*, and *NbUGT17* positively regulate CWMV infection in *N. benthamiana*.

## 4. Discussion

Glycosylation, a type of post-translational modification (PTM) of protein, is catalyzed by GTs and can affect several cellular processes and metabolic pathways in plants, including host–pathogen interactions [42,43,44]. GTs are a class of highly differentiated multi-membered metabolic enzymes belonging to the multi-gene transferase family. Based on the similarity of amino acid sequences and substrate specificity, they are divided into 117 families (CAZy, http://www.cazy.org, accessed on 3 December 2023), among which the GT1 family has the most members and is the most functionally important. The GT1 family mainly catalyzes UDP-glucides to specific receptors, so it is also commonly referred to as the UGT family [45]. The crystal structures of plant UGTs exhibit GT-B folding, which is a common structural folding of nucleotide sugar-dependent enzymes [6,46]. The GT-B folding consists of two flexibly connected β/α/β Roseman domains. The gap between these two domains is critical for the catalytic activity [6,47]. The two domains are referred to as the C-terminal and N-terminal domains. Its C-terminal domain recognizes and binds UDP-glucides through a highly conserved 44 amino acid motif known as the PSPG motif, which is the basis of this study; the N-terminal domain binds aglycones loosely, resulting in substrate structural diversity [48,49,50].

In addition, the implementation of the Human Genome Project (HGP) and the rapid development of high-throughput sequencing technology have provided a vast amount of data for bioinformatics development. UGT genes have been identified and analyzed in many plants, such as maize, cotton, and tomato [51,52,53]. *N. benthamiana* is a heterotetraploid species with 19 chromosomes. It is known for small plants, luxuriant leaves, and is easy to cultivate. Moreover, *N. benthamiana* is susceptible to many pathogenic microorganisms, particularly viruses, and is subjected to gene expression regulation and PTMs in vivo. Therefore, it is commonly used as a model plant in biology. This study presented a genome-wide analysis of the UGT family in *N. benthamiana* and investigated the response of some NbUGT genes to CWMV. Based on the PSPG motif, 147 UGT genes have been identified in *N. benthamiana*. These genes were divided into 16 groups using cluster analysis. Among them, Group A had the largest number of genes, with 25 genes accounting for 17.00% of the UGT genes in *N. benthamiana*. In addition, there were two new groups, Group O and Group P, which were specific to *Arabidopsis thaliana* and included 24 and 12 genes, respectively (Figure 1). These two groups have been found in wheat, as well [54]. In *N. benthamiana*, 81.63% of the NbUGTs did not contain any introns or contained only one intron, which is consistent with the number of UGT gene introns in tomato, rice, and cotton [51,53,55]. In this study, the website (http://cello.life.nctu.edu.tw/cello.html, accessed on 16 September 2022) was used to predict that these NbUGT genes were primarily located in the cytoplasm, whereas the UGT genes of wheat were mostly located in the cytoplasm, cell membrane, and chloroplast [54], and the UGT genes of cotton were mostly located in the cytosol and chloroplast [56]. Out of the 147 UGT genes, 92.52% had CDS lengths between 1000 and 1500 bp, and 89.80% were acidic proteins (pI < 7). Moreover, three genes, *NbUGT12*, *NbUGT16*, and *NbUGT17*, were found to be acidic proteins containing only one intron (Table 1).

*Bronze1*, the first gene in the plant UGT family, was accidentally discovered in 1977. The protein encoded by *Bronze1* is an enzyme with UGT activity that synthesizes flavonoid glycosides and regulates melanin accumulation in maize grains [57]. Since then, numerous studies have been conducted on plant UGT genes, particularly in food crops, medicinal plants, fruits, and flavonoid-rich plants such as *Rhodiola sachalinensis*, *Ginkgo biloba*, and strawberry. However, most reports focused on the identification of UGT genes and their role in plant growth and development, fruit quality, or adaptation to abiotic stress [58,59,60,61,62,63,64]. In-depth studies have been conducted on some model crops, such as rice, *Arabidopsis thaliana*, tomato, and the phenomenon and mechanism of some UGT genes responding to pathogens have been elucidated [55,65,66]. However, the relationship between most UGT genes in plants and pathogens, especially viruses, remains unclear.

The expression levels of eight *NbUGTs* were upregulated significantly during CWMV infection (Figure 4). These genes may play a role in the interaction between *N. benthamiana* and CWMV, which could be necessary for CWMV infection or the host response to it. Further analysis revealed that *NbUGT12*, *NbUGT16*, and *NbUGT17* were susceptible genes that positively regulated CWMV invasion (Figure 6). Interestingly, *NbUGT12*-silenced *N. benthamiana* plants showed a significant dwarf phenotype, suggesting the potential role that *NbUGT12* may play in CWMV–host interactions, as well as plant growth and development. A similar phenomenon has been observed in *Arabidopsis thaliana*, where *UGT73C7* contributes to plant disease resistance, but the overexpression lines of *UGT73C7* show a dwarf phenotype [66].

The subcellular localization of NbUGT12, NbUGT16, and NbUGT17 remained unchanged during CWMV infection (Figure 5), indicating that they should not affect viral infection through changes in localization. Many UGT genes are involved in the phenylpropanoid metabolism in plants, which can regulate the accumulation of metabolites such as hydroxycinnamic acids, coumarins, flavonoids, phenols, and tannins [66,67,68,69,70]. Flavonoid glycosylation is typically mediated by UGTs [1]. It provides the core flavonoid skeleton with complexity, enhances molecular stability and solubility, alters its chemical properties, and affects subcellular transport and biological activity [10,70,71]. The phenylpropanoid metabolism also regulates plant growth and development [72,73]. Therefore, it is possible that these three UGT genes, particularly *NbUGT12*, are involved in the flavonoid metabolism or other links in the phenylpropanoid metabolic pathway, which could affect plant growth and susceptibility to CWMV infection. Some UGTs are also involved in hormone pathways, such as JA, SA, and abscisic acid (ABA) [23,74,75,76], which are also related to plant resistance. For instance, it was discovered that aspirin (acetyl-SA) was resistant to tobacco mosaic virus in 1979, marking the first report of SA’s involvement in plant immunity [77]. Subsequently, numerous studies have demonstrated that SA is a defense-related hormone [78,79,80]. Additionally, plant immunity is highly dependent on the interaction between SA and other hormones [81,82]. The balance between plant growth and immunity is also influenced by SA [83]. Whether *NbUGT12*, *NbUGT16*, and *NbUGT17* are associated with these hormone pathways or the phenylpropanoid metabolism, and whether they participate in singular or multiple pathways to synergistically affect the interaction between *N. benthamiana* and CWMV, are directions for future studies.

## 5. Conclusions

Accumulating evidence suggests that glycosylation, as a common PTM, is essential for growth, development, and immunity in eukaryotes. However, to date, there have been very few instances concerning the study of the functions and mechanisms of glycosylation in the interaction between pathogens, especially viruses and their hosts in plants. In this study, we identified and analyzed 147 UGT genes in *N. benthamiana* via bioinformatics methods. Bioinformatics analysis, such as the physicochemical information, gene structure, conserved motifs, and conserved domains, showed that the UGT gene family is highly conserved in *N. benthamiana*. More importantly, the expression patterns showed that eight NbUGT genes may be involved in CWMV infection. The function of three genes, *NbUGT12*, *NbUGT16*, and *NbUGT17*, which positively regulate CWMV infection has been initially confirmed by transient overexpression and VIGS assays. These findings could facilitate the investigation of the molecular mechanisms of the interplay between CWMV and its natural host wheat, which can contribute to breeding resistant varieties to CWMV.

## Figures and Tables

**Table 1:** List of physicochemical properties and subcellular localization of NbUGTs.

Gene Stable ID/Locus Name	MW (kDa)	PL	pI	CDS Length/bp	IN	SL
Niben101Scf00173g06003.1	55.25088	489	6.01	1470	0	Cytoplasmic PlasmaMembrane
Niben101Scf00173g06007.1	40.97301	359	6.94	1080	4	Cytoplasmic
Niben101Scf00175g01002.1	36.15161	315	5.61	948	0	PlasmaMembrane
Niben101Scf00175g02009.1	35.93907	319	4.92	960	1	Cytoplasmic
Niben101Scf00175g02019.1	53.26710	475	5.16	1428	1	Cytoplasmic
Niben101Scf00270g15011.1	53.74701	475	5.49	1428	1	PlasmaMembrane
Niben101Scf00355g04002.1	52.24085	464	5.32	1395	0	Cytoplasmic
Niben101Scf00492g00010.1	38.22123	336	7.09	1011	0	Cytoplasmic
Niben101Scf00503g01003.1	52.50974	478	5.43	1437	0	Cytoplasmic
Niben101Scf00539g02028.1	52.55631	458	7.43	1377	0	Cytoplasmic
Niben101Scf00560g05006.1	53.88507	480	6.77	1443	0	PlasmaMembrane
Niben101Scf00661g00002.1	51.93085	463	6.93	1392	1	Chloroplast Cytoplasmic
Niben101Scf00669g00008.1	55.45511	487	5.77	1464	0	Cytoplasmic
Niben101Scf00672g01002.1	57.90343	510	6.37	1533	1	Cytoplasmic PlasmaMembrane
Niben101Scf00788g02013.1	51.60161	457	5.37	1374	1	Cytoplasmic
Niben101Scf00788g02014.1	63.31325	561	5.77	1686	1	Cytoplasmic
Niben101Scf00788g02015.1	56.78464	500	5.53	1503	1	PlasmaMembrane Cytoplasmic Chloroplast
Niben101Scf00817g06013.1	53.06923	479	5.68	1440	1	Cytoplasmic
Niben101Scf00906g00015.1	53.08143	479	6.50	1440	1	Chloroplast Cytoplasmic
Niben101Scf01017g03006.1	50.54148	449	4.69	1350	1	Cytoplasmic
Niben101Scf01124g24003.1	51.36213	454	5.38	1365	0	Cytoplasmic
Niben101Scf01188g08019.1	50.64306	457	6.37	1374	1	Cytoplasmic
Niben101Scf01225g02009.1	43.94214	390	6.48	1173	2	Cytoplasmic
Niben101Scf01225g02010.1	46.67563	418	4.83	1257	1	Cytoplasmic
Niben101Scf01300g02011.1	43.07863	379	6.63	1140	0	Chloroplast
Niben101Scf01300g04001.1	52.21414	464	5.02	1395	0	Cytoplasmic
Niben101Scf01341g00002.1	56.11798	503	6.08	1512	0	Cytoplasmic
Niben101Scf01386g00005.1	53.27002	472	6.08	1419	0	Cytoplasmic PlasmaMembrane
Niben101Scf01390g01004.1	55.40395	488	6.45	1467	0	PlasmaMembrane Cytoplasmic
Niben101Scf01409g03002.1	49.66975	452	4.88	1359	1	Cytoplasmic
Niben101Scf01494g06002.1	32.08006	282	5.25	849	3	Cytoplasmic
Niben101Scf01557g05005.1	69.25793	616	6.18	1851	1	Cytoplasmic
Niben101Scf01557g06005.1	49.85595	441	6.53	1326	1	Cytoplasmic
Niben101Scf01559g01014.1	52.66307	469	5.86	1410	0	Cytoplasmic
Niben101Scf01634g07029.1	55.20606	487	7.86	1464	1	Cytoplasmic Mitochondrial
Niben101Scf01660g00007.1	52.94962	471	5.15	1416	0	Cytoplasmic
Niben101Scf01660g02013.1	53.50339	473	6.01	1422	0	Cytoplasmic PlasmaMembrane
Niben101Scf01660g02014.1	54.15285	481	5.61	1446	0	PlasmaMembrane
Niben101Scf01763g00016.1	50.55943	445	5.84	1338	0	Cytoplasmic
Niben101Scf01777g03010.1	50.47786	448	6.63	1347	0	Chloroplast Cytoplasmic
Niben101Scf01777g03029.1	43.98762	390	7.11	1173	2	Cytoplasmic Mitochondrial
Niben101Scf01795g02015.1	41.70329	370	6.58	1113	3	Cytoplasmic Nuclear
Niben101Scf01834g04027.1	70.89713	619	6.19	1860	3	Cytoplasmic
Niben101Scf01951g00028.1	51.00416	454	5.93	1365	1	PlasmaMembrane Cytoplasmic
Niben101Scf01980g10004.1	54.34053	485	5.23	1458	1	Cytoplasmic
Niben101Scf01998g03011.1	56.29511	494	6.37	1485	1	Cytoplasmic
Niben101Scf01999g03009.1	56.56114	496	5.49	1491	0	Cytoplasmic
Niben101Scf02085g18002.1	54.48497	481	5.11	1446	1	Cytoplasmic
Niben101Scf02139g02011.1	56.47553	497	6.47	1494	1	Cytoplasmic
Niben101Scf02315g01007.1	56.55771	498	6.35	1497	1	Cytoplasmic
Niben101Scf02399g02004.1	53.54442	468	4.94	1407	0	Cytoplasmic
Niben101Scf02405g04013.1	55.28666	485	7.44	1458	3	Cytoplasmic
Niben101Scf02413g04005.1	47.04465	411	6.62	1236	1	Cytoplasmic
Niben101Scf02437g02019.1	51.83977	460	5.37	1383	1	OuterMembrane Cytoplasmic
Niben101Scf02476g03009.1	56.22729	502	5.14	1509	0	Cytoplasmic OuterMembrane
Niben101Scf02476g03010.1	57.12081	507	6.15	1524	0	Cytoplasmic
Niben101Scf02502g08002.1	54.26894	494	6.38	1485	0	OuterMembrane Cytoplasmic
Niben101Scf02537g08001.1	54.44675	485	5.42	1458	1	Cytoplasmic
Niben101Scf02537g09004.1	55.60343	495	5.72	1488	1	Cytoplasmic
Niben101Scf02562g02012.1	42.27469	376	5.59	1131	1	Cytoplasmic
Niben101Scf02565g03002.1	53.48191	478	5.63	1437	1	Cytoplasmic
Niben101Scf02606g05025.1	45.87567	401	5.95	1206	5	PlasmaMembrane
Niben101Scf02653g06007.1	53.47655	474	5.28	1425	1	PlasmaMembrane Cytoplasmic
Niben101Scf02751g02006.1	69.02013	612	5.37	1839	3	Cytoplasmic
Niben101Scf02752g10002.1	50.66152	449	6.34	1350	0	PlasmaMembrane Cytoplasmic Chloroplast
Niben101Scf02807g01001.1	53.25338	475	5.37	1428	0	Chloroplast
Niben101Scf02807g02003.1	49.35174	443	5.11	1332	2	Cytoplasmic
Niben101Scf02807g03003.1	43.19847	387	5.21	1164	2	Cytoplasmic Chloroplast
Niben101Scf02807g03005.1	53.50874	482	5.50	1449	0	Cytoplasmic
Niben101Scf02941g00009.1	51.88057	453	7.60	1362	0	Cytoplasmic Mitochondrial Nuclear
Niben101Scf03012g03021.1	51.94987	460	5.27	1383	1	Cytoplasmic
Niben101Scf03046g04015.1	91.45533	801	6.10	2406	4	Cytoplasmic
Niben101Scf03056g02018.1	49.93344	454	5.11	1365	1	PlasmaMembrane
Niben101Scf03108g10001.1	53.36225	466	5.09	1401	0	Cytoplasmic
Niben101Scf03108g14001.1	53.57633	467	6.12	1404	1	Cytoplasmic
Niben101Scf03223g00003.1	55.78762	492	6.43	1479	0	Cytoplasmic
Niben101Scf03427g10003.1	50.63953	450	6.14	1353	0	Cytoplasmic
Niben101Scf03434g01013.1	55.40669	493	5.77	1482	0	Cytoplasmic
Niben101Scf03438g02007.1	50.72439	449	6.63	1350	0	Cytoplasmic
Niben101Scf03536g01017.1	48.24982	429	6.51	1290	1	Cytoplasmic
Niben101Scf03607g01008.1	40.13816	358	5.06	1077	0	Cytoplasmic
Niben101Scf03709g03002.1	55.30632	496	5.40	1491	1	Cytoplasmic
Niben101Scf03779g08020.1	53.47131	470	5.46	1413	1	Cytoplasmic
Niben101Scf03929g05003.1	31.39917	280	5.54	843	3	Cytoplasmic
Niben101Scf03973g00012.1	62.75820	559	7.52	1680	5	Mitochondrial Cytoplasmic
Niben101Scf03983g00029.1	51.55136	453	6.14	1362	0	Cytoplasmic
Niben101Scf04007g02018.1	55.76853	492	6.33	1479	0	Cytoplasmic
Niben101Scf04187g01002.1	51.39185	467	6.53	1404	0	Cytoplasmic
Niben101Scf04240g00006.1	49.98282	449	6.17	1350	1	Mitochondrial Chloroplast Cytoplasmic
Niben101Scf04296g00015.1	44.93744	395	7.50	1188	1	PlasmaMembrane
Niben101Scf04404g01009.1	52.33412	465	8.97	1398	0	Chloroplast Mitochondrial
Niben101Scf04404g03001.1	52.62037	464	9.76	1395	1	PlasmaMembrane
Niben101Scf04871g08010.1	52.96204	476	5.68	1431	0	Cytoplasmic Chloroplast
Niben101Scf04871g08011.1	52.55898	468	6.30	1407	0	Cytoplasmic
Niben101Scf04871g08015.1	49.54030	448	6.70	1347	1	Cytoplasmic Chloroplast
Niben101Scf04875g02008.1	50.66160	450	4.83	1353	1	Cytoplasmic
Niben101Scf04940g01021.1	53.47241	481	5.54	1446	0	PlasmaMembrane
Niben101Scf04967g00002.1	30.55219	270	7.13	813	2	Cytoplasmic
Niben101Scf05300g02015.1	39.04028	342	4.95	1029	3	PlasmaMembrane
Niben101Scf05307g00001.1	48.83847	443	5.93	1332	0	Cytoplasmic Chloroplast PlasmaMembrane
Niben101Scf05308g01008.1	50.29414	452	5.46	1359	2	Cytoplasmic
Niben101Scf05415g00003.1	51.39926	456	5.46	1371	1	PlasmaMembrane Cytoplasmic
Niben101Scf06112g01008.1	53.47046	477	5.97	1434	1	Cytoplasmic
Niben101Scf06233g01011.1	50.64607	454	6.34	1365	1	PlasmaMembrane
Niben101Scf06344g00005.1	52.51495	466	5.31	1401	0	Cytoplasmic
Niben101Scf06344g01003.1	41.46452	372	4.80	1119	0	Cytoplasmic
Niben101Scf06374g00013.1	50.73411	450	6.50	1353	1	PlasmaMembrane
Niben101Scf06374g00014.1	43.93319	390	6.65	1173	4	PlasmaMembrane
Niben101Scf06388g03004.1	53.42717	481	6.25	1446	0	Cytoplasmic
Niben101Scf06408g04004.1	51.19207	454	4.87	1365	1	Cytoplasmic Nuclear
Niben101Scf06846g04002.1	49.89490	443	6.31	1332	0	Cytoplasmic
Niben101Scf06942g02006.1	51.59511	457	6.20	1374	1	Cytoplasmic
Niben101Scf07089g00006.1	53.24665	471	8.53	1416	0	Cytoplasmic Mitochondrial
Niben101Scf07089g02004.1	52.01572	463	5.61	1392	0	Chloroplast PlasmaMembrane Cytoplasmic
Niben101Scf07226g08002.1	46.71687	411	6.61	1236	3	PlasmaMembrane
Niben101Scf07325g00029.1	47.18517	430	5.98	1293	3	PlasmaMembrane
Niben101Scf07353g02006.1	49.38242	442	6.55	1329	0	Cytoplasmic Mitochondrial
Niben101Scf07563g02008.1	52.72471	471	6.09	1416	0	Cytoplasmic
Niben101Scf07585g00007.1	37.81300	334	6.03	1005	2	PlasmaMembrane Cytoplasmic
Niben101Scf08015g03004.1	51.62843	454	5.53	1365	0	Cytoplasmic PlasmaMembrane
Niben101Scf08249g00001.1	52.34963	459	6.36	1380	1	Nuclear Cytoplasmic PlasmaMembrane
Niben101Scf08467g03008.1	60.48347	530	5.98	1593	1	Cytoplasmic Nuclear
Niben101Scf08549g02008.1	55.34183	490	5.31	1473	1	PlasmaMembrane
Niben101Scf08835g00007.1	54.52800	483	6.48	1452	1	PlasmaMembrane Cytoplasmic Mitochondrial
Niben101Scf09184g01003.1	46.08446	405	6.05	1218	0	Cytoplasmic
Niben101Scf09225g04002.1	50.10597	446	6.23	1341	1	Cytoplasmic
Niben101Scf09822g01016.1	42.25115	376	5.79	1131	1	Cytoplasmic
Niben101Scf11008g01002.1	53.83543	475	6.30	1428	0	Cytoplasmic
Niben101Scf11008g03003.1	52.19999	464	8.58	1395	1	PlasmaMembrane
Niben101Scf11183g00001.1	55.52863	491	6.48	1476	0	Cytoplasmic PlasmaMembrane
Niben101Scf12290g01008.1	52.29987	458	6.16	1377	0	PlasmaMembrane
Niben101Scf12919g00005.1	53.55560	476	6.43	1431	0	Cytoplasmic
Niben101Scf12919g00008.1	51.96516	465	4.83	1398	0	Cytoplasmic
Niben101Scf12919g00023.1	43.26769	381	6.20	1146	0	Cytoplasmic
Niben101Scf12919g00035.1	41.70371	369	7.23	1110	5	Cytoplasmic Chloroplast
Niben101Scf13710g02002.1	54.38508	488	5.78	1467	0	Cytoplasmic
Niben101Scf14769g01001.1	48.72355	434	7.28	1305	3	Cytoplasmic Mitochondrial
Niben101Scf14996g00010.1	53.55044	480	6.47	1443	1	PlasmaMembrane
Niben101Scf15817g01009.1	51.89515	460	5.21	1383	0	Cytoplasmic
Niben101Scf17597g01013.1	37.80270	336	5.01	1011	0	Cytoplasmic Chloroplast
Niben101Scf17612g02009.1	50.56254	444	6.22	1335	0	Cytoplasmic
Niben101Scf18348g01035.1	54.45771	488	5.11	1467	2	PlasmaMembrane Cytoplasmic
Niben101Scf22015g01001.1	49.64530	449	6.96	1350	2	Chloroplast Cytoplasmic
Niben101Scf27793g00001.1	82.62507	727	5.34	2184	2	Cytoplasmic PlasmaMembrane
Niben101Scf28267g00010.1	42.14358	379	5.01	1140	0	Cytoplasmic
Niben101Scf28267g00014.1	44.16199	394	6.58	1185	2	Cytoplasmic
Niben101Scf32539g00003.1	49.55187	440	6.52	1323	1	PlasmaMembrane

PL, protein length; pI, isoelectric point; MW, molecular weight; CDS, coding sequence; IN, intron number; SL, subcellular location.

## Data Availability

Data are contained within the article and Supplementary Materials.

## Supplementary Materials

The following supporting information can be downloaded at: https://www.mdpi.com/article/10.3390/v16040489/s1, Table S1: Amino acid sequences of ten conserved motifs; Table S2: Nucleotide sequences of silent fragments for *NbUGT12*, *NbUGT16*, and *NbUGT17*; Table S3: All primer sequences used in this study; Table S4: The corresponding genes’ names in this study; Figure S1: The expression patterns of 12 *NbUGTs* without significant changes under CWMV infection; Figure S2: Detection of the presence of introns for *NbUGT12*, *NbUGT16*, and *NbUGT17*.
