# Supplementary material for: Genome-Wide Analysis and Identification of UDP Glycosyltransferases Responsive to Chinese Wheat Mosaic Virus Resistance in Nicotiana benthamiana
Source: Viruses. 2024 Mar 22;16(4):489. doi: 10.3390/v16040489 (PMC11054786; doi:10.3390/v16040489)
Supplement: Supplementary file 1 [file viruses-16-00489-s001.zip › viruses-2868224-supplementary/Supplementary File-viruses-2868224/Figure S1.pdf]

SUPPORTING INFORMATION:

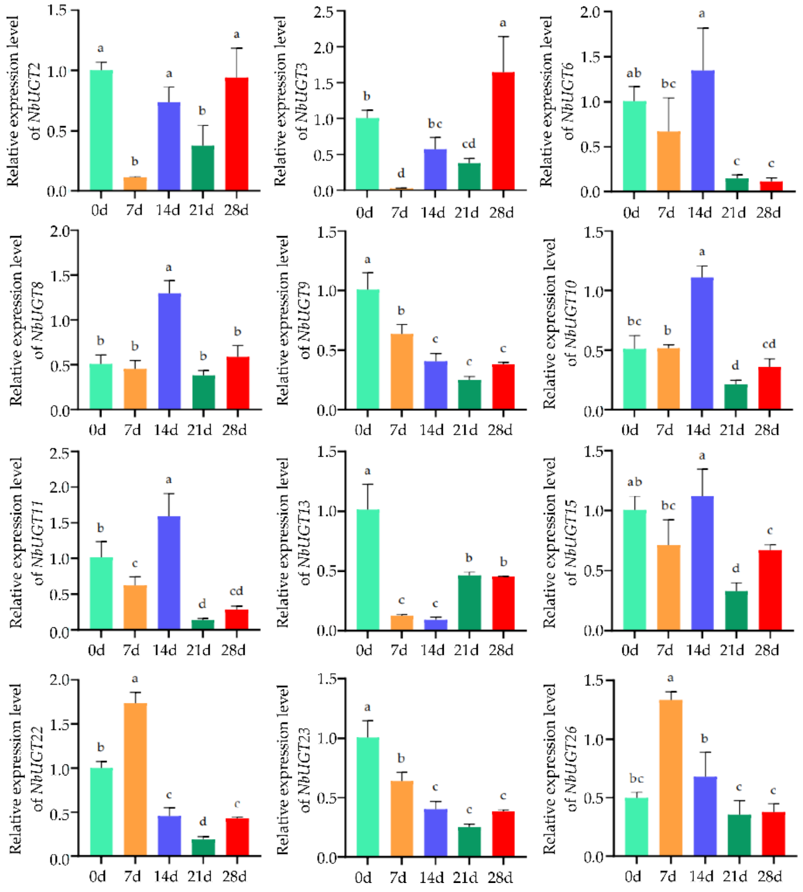

Figure S1. The expression patterns of 12 UGT genes in CWMV-infected *N. benthamiana* plants. Relative expression levels of *NbUGT2*, *NbUGT3*, NbUGT6, *NbUGT8*, *NbUGT9*, *NbUGT10*, *NbUGT11*, *NbUGT13*, *NbUGT15*, *NbUGT22*, *NbUGT23*, and *NbUGT26* in CWMV-infected *N. benthamiana* plants at 0 to 28 dpi. Each treatment had three biological replicates, the data presented are the means ± SD, determined using the Student's *t*-test. Different letters show statistically significant differences ($p < 0.05$, Tukey's test).
