# Supplementary material for: Genome-Wide Analysis and Identification of UDP Glycosyltransferases Responsive to Chinese Wheat Mosaic Virus Resistance in Nicotiana benthamiana
Source: Viruses. 2024 Mar 22;16(4):489. doi: 10.3390/v16040489 (PMC11054786; doi:10.3390/v16040489)
Supplement: Supplementary file 1 [file viruses-16-00489-s001.zip › viruses-2868224-supplementary/Supplementary File-viruses-2868224/Figure S2.pdf]

```
NbUGT12-cDNA.seq   AATTCTTCAATGTTTTTGTCTGAACTCCCTCCTTCATCCACAGCATTTCTAGCAAATTCCTTCCATTTCTTTGCATTTTC     80
NbUGT12-DNA.seq    AATTCTTCAATGTTTTTGTCTGAACTCCCTCCTTCATCCACAGCATTTCTAGCAAATTCCTTCCATTTCTTTGCATTTTC     80
Consensus          aattcttcaatgtttttgtctgaactccctccttcatccacagcatttctagcaaattccttccatttctttgcattttc

NbUGT12-cDNA.seq   CCTAATTACTTTTCCTTTCTCTTCTTCCATCACTAATTTTATATTTTCTTCAATAATTTCTCTTCTAACTATTCCTTTTT    160
NbUGT12-DNA.seq    CCTAATTACTTTTCCTTTCTCTTCTTCCATCACTAATTTTATATTTTCTTCAATAATTTCTCTTCTAACTATTCCTTTTT    160
Consensus          cctaattacttttcctttctcttcttccatcactaattttatattttcttcaataatttctcttctaactattccttttt

NbUGT12-cDNA.seq   CATCTTGTTTGGCTCTAACTCCCATCTCCCAAACATCCTGCACAAGCTTTGCATTTGTTGGTTGATCCGACCATTGGGGC    240
NbUGT12-DNA.seq    CATCTTGTTTGGCTCTAACTCCCATCTCCCAAACATCCTGCACAAGCTTTGCATTTGTTGGTTGATCCGACCATTGGGGC    240
Consensus          catcttgtttggctctaactcccatctcccaaacatcctgcacaagctttgcatttgttggttgatccgaccattggggc

NbUGT12-cDNA.seq   ATTGTCACTATTGGCACTCCCAAACTAATTGCTTCTAAAGTCGAATTCCATCCACTATGTGTAAGAAAACACCCTATCGA    320
NbUGT12-DNA.seq    ATTGTCACTATTGGCACTCCCAAACTAATTGCTTCTAAAGTCGAATTCCATCCACTATGTGTAAGAAAACACCCTATCGA    320
Consensus          attgtcactattggcactcccaaactaattgcttctaaagtcgaattccatccactatgtgtaagaaaacaccctatcga

NbUGT12-cDNA.seq   TTTATGTTCCAACACTTGTAATTGGGGACACCATGAAACCACGAAACCTTTTTTCTCACTTGCTGATTTTAATTCCTCTA    400
NbUGT12-DNA.seq    TTTATGTTCCAACACTTGTAATTGGGGACACCATGAAACCACGAAACCTTTTTTCTCACTTGCTGATTTTAATTCCTCTA    400
Consensus          tttatgttccaacacttgtaattggggacaccatgaaaccacgaaaccttttttctcacttgctgattttaattcctcta

NbUGT12-cDNA.seq   TAAAGTTCTTGGGAAGTTTGGATTCTTCAGTGGATCTAACCACCCACAAAAAGTTCTTGTTGCTATTCTTCGAACCCCAA    480
NbUGT12-DNA.seq    TAAAGTTCTTGGGAAGTTTGGATTCTTCAGTGGATCTAACCACCCACAAAAAGTTCTTGTTGCTATTCTTCGAACCCCAA    480
Consensus          taaagttcttgggaagtttggattcttcagtggatctaaccacccacaaaaagttcttgttgctattcttcgaaccccaa

NbUGT12-cDNA.seq   GCCAATTCTTCCATTTGCTCAGCTTCTAATTTAGCCATACTTCCAAATGATACATACACTACTGAGCTAATTGGTTGAGG    560
NbUGT12-DNA.seq    GCCAATTCTTCCATTTGCTCAGCTTCTAATTTAGCCATACTTCCAAATGATACATACACTACTGAGCTAATTGGTTGAGG    560
Consensus          gccaattcttccatttgctcagcttctaatttagccatacttccaaatgatacatacactactgagctaattggttgagg

NbUGT12-cDNA.seq   ATTCAACCAATTTAAGCACTCTTTTGCCATCGGCTTGAAGAGACTAAGGCCATACTCTTTGTCATTTGGTAGCCTCTTGT    640
NbUGT12-DNA.seq    ATTCAACCAATTTAAGCACTCTTTTGCCATCGGCTTGAAGAGACTAAGGCCATACTCTTTGTCATTTGGTAGCCTCTTGT    640
Consensus          attcaaccaatttaagcactcttttgccatcggcttgaagagactaaggccatactctttgtcatttggtagcctcttgt

NbUGT12-cDNA.seq   CTAGGTACATGGATGGTATTGTTGGTCCAATTGTCTTGATTGGATAAAACTTGGCCATCCAATCAA..............    706
NbUGT12-DNA.seq    CTAGGTACATGGATGGTATTGTTGGTCCAATTGTCTTGATTGGATAAAACTTGGCCATCCAATCAATTACCTACATGTCT    720
Consensus          ctaggtacatggatggtattgttggtccaattgtcttgattggataaaacttggccatccaatcaa

NbUGT12-cDNA.seq   ................................................................................    706
NbUGT12-DNA.seq    AGCAATTAGAAACATGATAATCTAAATACACTGACAATGTAAATGACATCTTTTTATGTTACTTATTAGTGCAGTTTAGC    800
Consensus

NbUGT12-cDNA.seq   ................................................................................    706
NbUGT12-DNA.seq    ATGTGATAGTAGGTCCATATTGTATCTTCTTAAAGGTTTTACACGTGCTTTAATAAAGATATTTAATATCCCTAATTATA    880
Consensus

NbUGT12-cDNA.seq   ................................................................................    706
NbUGT12-DNA.seq    GGAGAGTACTCGTATAAACTACTTCTTTGTAAACGTTTCGCTTAATGAATATTCTGCTCCAACAATATGGTAAGCTTGAA    960
Consensus

NbUGT12-cDNA.seq   ................................................................................    706
NbUGT12-DNA.seq    AACAAGGTGAGAAATGTTTTTTTTTTGGTGTTTTTTGAAACGTTAAATATTACGAAAGAAATCCAATTTGTGATACAATT   1040
Consensus

NbUGT12-cDNA.seq   ................................................................................    706
NbUGT12-DNA.seq    ACTCCTACAAATTTTGGAATCTGGATGAGAACTGAGAATAAGATATATGGATTTAATAGACAAAGCAAAAGAAAAGAAGT   1120
Consensus

NbUGT12-cDNA.seq   ...............TTACCTCTTTCTCCAACTCATAGAAGCTGTTGATTAGGACCCAATCCACTTTCTCAAGATTTGAG    771
NbUGT12-DNA.seq    AGAAGAATATTCCTCTTACCTCTTTCTCCAACTCATAGAAGCTGTTGATTAGGACCCAATCCACTTTCTCAAGATTTGAG   1200
Consensus                         ttacctctttctccaactcatagaagctgttgattaggacccaatccactttctcaagatttgag

NbUGT12-cDNA.seq   AATTGATTCACCAACATTTCAACTAATTTATCTGATTGAGGACTAGACTCAAAACTAGGTACATCTGAACTCTCAATTGT    851
NbUGT12-DNA.seq    AATTGATTCACCAACATTTCAACTAATTTATCTGATTGAGGACTAGACTCAAAACTAGGTACATCTGAACTCTCAATTGT   1280
Consensus          aattgattcaccaacatttcaactaatttatctgattgaggactagactcaaaactaggtacatctgaactctcaattgt

NbUGT12-cDNA.seq   ACTTGATAATCCAGGAATTAATATTTGTCCATCAACTTGAGTAGGAGGAAGTTTTAGTACCCCTTTATGTACATGGTAGT    931
NbUGT12-DNA.seq    ACTTGATAATCCAGGAATTAATATTTGTCCATCAACTTGAGTAGGAGGAAGTTTTAGTACCCCTTTATGTACATGGTAGT   1360
Consensus          acttgataatccaggaattaatatttgtccatcaacttgagtaggaggaagttttagtacccctttatgtacatggtagt

NbUGT12-cDNA.seq   AAATGTTATCTACTACACAATTTTGTGTGAAAAAAGCAGCATTAACTAATCCAAGATCCTTCGCTACTTCAACAGCCCAA   1011
NbUGT12-DNA.seq    AAATGTTATCTACTACACAATTTTGTGTGAAAAAAGCAGCATTAACTAATCCAAGATCCTTCGCTACTTCAACAGCCCAA   1440
Consensus          aaatgttatctactacacaattttgtgtgaaaaaagcagcattaactaatccaagatccttcgctacttcaacagcccaa

NbUGT12-cDNA.seq   GGAAGGAATGGATCATAAACTATGCAATTCACAGGGTACTCACAACTTTCTAATTTCTTAATAAGTTGAGTCAGAGTATC   1091
NbUGT12-DNA.seq    GGAAGGAATGGATCATAAACTATGCAATTCACAGGGTACTCACAACTTTCTAATTTCTTAATAAGTTGAGTCAGAGTATC   1520
Consensus          ggaaggaatggatcataaactatgcaattcacagggtactcacaactttctaatttcttaataagttgagtcagagtatc

NbUGT12-cDNA.seq   GGAGCCAACTTCTTTGAATCGTGTTATGTAGGCCAAGAAAGATTCTGCTTGGTCGATGCCACCATCGTCGTAGCCATCGG   1171
NbUGT12-DNA.seq    GGAGCCAACTTCTTTGAATCGTGTTATGTAGGCCAAGAAAGATTCTGCTTGGTCGATGCCACCATCGTCGTAGCCATCGG   1600
Consensus          ggagccaacttctttgaatcgtgttatgtaggccaagaaagattctgcttggtcgatgccaccatcgtcgtagccatcgg

NbUGT12-cDNA.seq   ATATGGCCTTGATTGACACTGAAGTTGGCAATTCTTGCATGGTTTTCAAGAAGGATTTTGTAGGTGATATTGTGATTTTA   1251
NbUGT12-DNA.seq    ATATGGCCTTGATTGACACTGAAGTTGGCAATTCTTGCATGGTTTTCAAGAAGGATTTTGTAGGTGATATTGTGATTTTA   1680
Consensus          atatggccttgattgacactgaagttggcaattcttgcatggttttcaagaaggattttgtaggtgatattgtgatttta

NbUGT12-cDNA.seq   ACACCTTTGGATTCTAAACGTTTGGAAAATTGGAGCATTGGGTTGATATGACCTTGCACTGGATATGGCAAGATCAAGCA   1331
NbUGT12-DNA.seq    ACACCTTTGGATTCTAAACGTTTGGAAAATTGGAGCATTGGGTTGATATGACCTTGCACTGGATATGGCAAGATCAAGCA   1760
Consensus          acacctttggattctaaacgtttggaaaattggagcattgggttgatatgaccttgcactggatatggcaagatcaagca

NbUGT12-cDNA.seq   GTGAGCTT                                                                           1339
NbUGT12-DNA.seq    GTGAGCTT                                                                           1768
Consensus          gtgagctt
```

```
NbUGT16-cDNA.seq     TGGCACAAGGCCATATGATACCTATGATCGACATAGCTCGATTATTAGCACAACGAGGTGTTATTATCACAATTCTTTTG     80
NbUGT16-DNA.seq      TGGCACAAGGCCATATGATACCTATGATCGACATAGCTCGATTATTAGCACAACGAGGTGTTATTATCACAATTCTTTTG     80
Consensus            tggcacaaggccatatgatacctatgatcgacatagctcgattattagcacaacgaggtgttattatcacaattcttttg

NbUGT16-cDNA.seq     ACACCATCAAATGGCAACAGGTTCAGTACAGTTATCGCCCGTGCTATAGAGATTGGACTTAAAATTCAAGTAATTGACCT    160
NbUGT16-DNA.seq      ACACCATCAAATGGCAACAGGTTCAGTACAGTTATCGCCCGTGCTATAGAGATTGGACTTAAAATTCAAGTAATTGACCT    160
Consensus            acaccatcaaatggcaacaggttcagtacagttatcgcccgtgctatagagattggacttaaaattcaagtaattgacct

NbUGT16-cDNA.seq     CTATTTTCCAAGCTCAGAAGCAGGGCTACCAGAAGGGTGCGAAAACTGCGACATGGTTCCATCTATAGATATGATGAAGA    240
NbUGT16-DNA.seq      CTATTTTCCAAGCTCAGAAGCAGGGCTACCAGAAGGGTGCGAAAACTGCGACATGGTTCCATCTATAGATATGATGAAGA    240
Consensus            ctattttccaagctcagaagcagggctaccagaagggtgcgaaaactgcgacatggttccatctatagatatgatgaaga

NbUGT16-cDNA.seq     ATTTCTTCCTTGCTACTCAGATGGTTGAATCGCAAGTAGTAGACTCGTTGCAAGAATTAAATCCATTACCAAGTTGTTTA    320
NbUGT16-DNA.seq      ATTTCTTCCTTGCTACTCAGATGGTTGAATCGCAAGTAGTAGACTCGTTGCAAGAATTAAATCCATTACCAAGTTGTTTA    320
Consensus            atttcttccttgctactcagatggttgaatcgcaagtagtagactcgttgcaagaattaaatccattaccaagttgttta

NbUGT16-cDNA.seq     ATTTCCGATATGTGTTTTCCTTGGACAACTAATGTTGCTAAAAAATTTAACATTCCTCGGATTGTTTTTCATGGGATGTG    400
NbUGT16-DNA.seq      ATTTCCGATATGTGTTTTCCTTGGACAACTAATGTTGCTAAAAAATTTAACATTCCTCGGATTGTTTTTCATGGGATGTG    400
Consensus            atttccgatatgtgttttccttggacaactaatgttgctaaaaaatttaacattcctcggattgtttttcatgggatgtg

NbUGT16-cDNA.seq     TAGCTTCTCTTTATTGTGTTTACACAATTTGAGAGAAGGGAAAGTGTTGGAAAATGCTAATTCTGATACTGAGTATTTTT    480
NbUGT16-DNA.seq      TAGCTTCTCTTTATTGTGTTTACACAATTTGAGAGAAGGGAAAGTGTTGGAAAATGCTAATTCTGATACTGAGTATTTTT    480
Consensus            tagcttctctttattgtgtttacacaatttgagagaagggaaagtgttggaaaatgctaattctgatactgagtattttt

NbUGT16-cDNA.seq     CAGTGCCTGGATTTCCAGATAAAGTTGAACTAACCAAAGCTCAACTTAAAACTTTGGTTGATCCAAGCAATCCTGAATGG    560
NbUGT16-DNA.seq      CAGTGCCTGGATTTCCAGATAAAGTTGAACTAACCAAAGCTCAACTTAAAACTTTGGTTGATCCAAGCAATCCTGAATGG    560
Consensus            cagtgcctggatttccagataaagttgaactaaccaaagctcaacttaaaactttggttgatccaagcaatcctgaatgg

NbUGT16-cDNA.seq     AATGAATTTGGGGAAAAAAATAAAGGAGGCAGAAGATGAAGCTTATGGTATAGTGGTGAATAGCTTTGAGGAGTTGGAACC    640
NbUGT16-DNA.seq      AATGAATTTGGGGAAAAAAATAAAGGAGGCAGAAGATGAAGCTTATGGTATAGTGGTGAATAGCTTTGAGGAGTTGGAACC    640
Consensus            aatgaatttggggaaaaaaataaaggaggcagaagatgaagcttatggtatagtggtgaatagctttgaggagttggaacc

NbUGT16-cDNA.seq     AGAATATGTAAAAAGATTGAAAAAGGCCAA..................................................    670
NbUGT16-DNA.seq      AGAATATGTAAAAAGATTGAAAAAGGCCAAAGGTAATGTTCAATTGCTTAAGTGTGTGATCGTCTTATCTGAACACTCAT    720
Consensus            agaatatgtaaaaagattgaaaaaggccaa

NbUGT16-cDNA.seq     ................................................................................    670
NbUGT16-DNA.seq      ACTGTCAGTAAATGCACACTTTTAATTAATTAATTATATCGATCATGTCTCAATGCGTAAGGGCCTAATTATTTTTTCAT    800
Consensus

NbUGT16-cDNA.seq     ................................................................................    670
NbUGT16-DNA.seq      GCATCAAACATGTCAAAATATTCAATATTCTTTGCTTTTGGATGGTGATAGAGATTTTAATTCACGCCCTCTGCTAAGTC    880
Consensus

NbUGT16-cDNA.seq     ................................................................................    670
NbUGT16-DNA.seq      CAATAACATGTTGAATTGTGTCACTATCTGATTTAAAAGTTTAACTAGAGGAAATACACTATTAATTGCGATATTATATT    960
Consensus

NbUGT16-cDNA.seq     ................................................................................    670
NbUGT16-DNA.seq      ATGTCACCGATGACCTCAACTTGCTTTAATATTATATTGAATTCCGTGAGCATCTTATCTAAAAGTTTAAGCTGTTACTG   1040
Consensus

NbUGT16-cDNA.seq     ................................................................................    670
NbUGT16-DNA.seq      AAAATACAAATTTTAATTGCAATAATTAAATGTGTCTCAAGCATATGTGGTTTGGTTCTATAAGCAAATGAAAATTTTCT   1120
Consensus

NbUGT16-cDNA.seq     ................................................................................    670
NbUGT16-DNA.seq      TTGCTTTAGGGGGTAGTGGTGAGATTTGAATTAAACCATGACGCATACTTGTTCTAATATCACATTGAATTGAGTGATCA   1200
Consensus

NbUGT16-cDNA.seq     .....................................................................AGGTAAGAAGA    681
NbUGT16-DNA.seq      TCTTATCTAAAAGCTTAAGTTATTAGAAGGAACATACTTTTAGTTACTAAAATATATAATGTCTCGAACAGGTAAGAAGA   1280
Consensus                                                                                 aggtaagaaga

NbUGT16-cDNA.seq     TTTGGACAATTGGTCCGGTTTCCTTATGCAACAAAGAGAAACAAGATAAAGCTGAAAGAGGAAGCAAAGCCTCTATAGAT    761
NbUGT16-DNA.seq      TTTGGACAATTGGTCCGGTTTCCTTATGCAACAAAGAGAAACAAGATAAAGCTGAAAGAGGAAGCAAAGCCTCTATAGAT   1360
Consensus            tttggacaattggtccggtttccttatgcaacaaagagaaacaagataaagctgaaagaggaagcaaagcctctatagat

NbUGT16-cDNA.seq     GAACATCACTGCTTGAAATGGCTCAATTCTAAGGAACCAGACTCTGTCCTCTTTGTTTGTCTTGGAAGTTTATCGCGCTT    841
NbUGT16-DNA.seq      GAACATCACTGCTTGAAATGGCTCAATTCTAAGGAACCAGACTCTGTCCTCTTTGTTTGTCTTGGAAGTTTATCGCGCTT   1440
Consensus            gaacatcactgcttgaaatggctcaattctaaggaaccagactctgtcctctttgtttgtcttggaagtttatcgcgctt

NbUGT16-cDNA.seq     GCCAGCATCACAGATGATAGAACTCGGACTTGCATTAGAGTCTTCTAAACGACCTTTTATTTGGGTTATTAGACATATTT    921
NbUGT16-DNA.seq      GCCAGCATCACAGATGATAGAACTCGGACTTGCATTAGAGTCTTCTAAACGACCTTTTATTTGGGTTATTAGACATATTT   1520
Consensus            gccagcatcacagatgatagaactcggacttgcattagagtcttctaaacgaccttttatttgggttattagacatattt

NbUGT16-cDNA.seq     CAGATGAACTCAAAAAAATGGTTAAATGAAGAGAATTTTGAAGAAAGAATTAAAGAACAAGGGATTTTAATCCATGGTTGG   1001
NbUGT16-DNA.seq      CAGATGAACTCAAAAAAATGGTTAAATGAAGAGAATTTTGAAGAAAGAATTAAAGAACAAGGGATTTTAATCCATGGTTGG   1600
Consensus            cagatgaactcaaaaaaatggttaaatgaagagaattttgaagaaagaattaaagaacaagggattttaatccatggttgg

NbUGT16-cDNA.seq     GCCCCACAAGTACTAATATTATCACATGCTTCTGTCGGGGGATTCGTGACTCATTGTGGATGGAATTCGAGTATAGAAGG   1081
NbUGT16-DNA.seq      GCCCCACAAGTACTAATATTATCACATGCTTCTGTCGGGGGATTCGTGACTCATTGTGGATGGAATTCGAGTATAGAAGG   1680
Consensus            gccccacaagtactaatattatcacatgcttctgtcgggggattcgtgactcattgtggatggaattcgagtatagaagg

NbUGT16-cDNA.seq     AATATCAACTGGCGTGCCAATGATCACTTGGCCATTATTTGCTGAGCAATTTTGTAATGAGAGGCTTATTACGAATGTTC   1161
NbUGT16-DNA.seq      AATATCAACTGGCGTGCCAATGATCACTTGGCCATTATTTGCTGAGCAATTTTGTAATGAGAGGCTTATTACGAATGTTC   1760
Consensus            aatatcaactggcgtgccaatgatcacttggccattatttgctgagcaattttgtaatgagaggcttattacgaatgttc

NbUGT16-cDNA.seq     TCAAGACAGGAGTAAAGTCTGGCGTCGAGAATCCTGTTATGTTTTTAGAGGAGGAAAAAGTGGATACTCAAGTGAACAAA   1241
NbUGT16-DNA.seq      TCAAGACAGGAGTAAAGTCTGGCGTCGAGAATCCTGTTATGTTTTTAGAGGAGGAAAAAGTGGATACTCAAGTGAACAAA   1840
Consensus            tcaagacaggagtaaagtctggcgtcgagaatcctgttatgtttttagaggaggaaaaagtggatactcaagtgaacaaa

NbUGT16-cDNA.seq     GATGACATTAAAATGGTTATTGAAAAATTAATGGGTGAAGAAGAGGAAGCAAAAATAAGAAGAGAAAGAGCTAAAAAGCT   1321
NbUGT16-DNA.seq      GATGACATTAAAATGGTTATTGAAAAATTAATGGGTGAAGAAGAGGAAGCAAAAATAAGAAGAGAAAGAGCTAAAAAGCT   1920
Consensus            gatgacattaaaatggttattgaaaaattaatgggtgaagaagaggaagcaaaaataagaagagaaagagctaaaaagct

NbUGT16-cDNA.seq     TGGAGAAATTGCAAGAAAGGCTGTGGAGGAAGGGGGTTCCTCTCACCTGAACTTGACAATACTAATACAAGA           1393
NbUGT16-DNA.seq      TGGAGAAATTGCAAGAAAGGCTGTGGAGGAAGGGGGTTCCTCTCACCTGAACTTGACAATACTAATACAAGA           1992
Consensus            tggagaaattgcaagaaaggctgtggaggaagggggttcctctcacctgaacttgacaatactaatacaaga
```

```
NbUGT17-cDNA.seq  GATGTCCCACCTTCATTAACAGCTTCCTTAGCCAAATCCTTCCATTTAAGGGCATTTTTCTTCATTTCTTTCCCCTTGTC    80
NbUGT17-DNA.seq   GATGTCCCACCTTCATTAACAGCTTCCTTAGCCAAATCCTTCCATTTAAGGGCATTTTTCTTCATTTCTTTCCCCTTGTC    80
Consensus         gatgtcccaccttcattaacagcttccttagccaaatccttccatttaagggcatttttcttcatttctttccccttgtc

NbUGT17-cDNA.seq  TCCTTCCATTACTATCCTTACACATTTCTCAATTTCTTCTCTTCCAGCAAACCCTTTCTCATTAACACTAACTCTCACCC   160
NbUGT17-DNA.seq   TCCTTCCATTACTATCCTTACACATTTCTCAATTTCTTCTCTTCCAGCAAACCCTTTCTCATTAACACTAACTCTCACCC   160
Consensus         tccttccattactatccttacacatttctcaatttcttctcttccagcaaaccctttctcattaacactaactctcaccc

NbUGT17-cDNA.seq  CTACACTCCAAACATCTTGTACTAATTTCGCATTTGTAGTTTGATCGGTCCATAGTGGCATTACAACCATCGGCACTCCA   240
NbUGT17-DNA.seq   CTACACTCCAAACATCTTGTACTAATTTCGCATTTGTAGTTTGATCGGTCCATAGTGGCATTACAACCATCGGCACTCCA   240
Consensus         ctacactccaaacatcttgtactaatttcgcatttgtagtttgatcggtccatagtggcattacaaccatcggcactcca

NbUGT17-cDNA.seq  AGACTCAAAGCTTCAGTCGTTGAATTCCATCCTCCATGTGAGAAAAACATCCCAATTGCCTTATTAGATAATATTTGTAA   320
NbUGT17-DNA.seq   AGACTCAAAGCTTCAGTCGTTGAATTCCATCCTCCATGTGAGAAAAACATCCCAATTGCCTTATTAGATAATATTTGTAA   320
Consensus         agactcaaagcttcagtcgttgaattccatcctccatgtgagaaaaacatcccaattgccttattagataatatttgtaa

NbUGT17-cDNA.seq  TTGTGGACTCCAATTTATTACTAATCCTTTCTCAGAGGTTTCCTCAATGAAATTTTTGGGAATTTTTGCTTCATCACAAG   400
NbUGT17-DNA.seq   TTGTGGACTCCAATTTATTACTAATCCTTTCTCAGAGGTTTCCTCAATGAAATTTTTGGGAATTTTTGCTTCATCACAAG   400
Consensus         ttgtggactccaatttattactaatcctttctcagaggtttcctcaatgaaatttttgggaatttttgcttcatcacaag

NbUGT17-cDNA.seq  TCCTAACAACCCATAAAAAGTAGTAATTGGTTGCTTTTAAACCCCAAGCTATTTCTTCCATTTGTTCATTATCCATAGTG   480
NbUGT17-DNA.seq   TCCTAACAACCCATAAAAAGTAGTAATTGGTTGCTTTTAAACCCCAAGCTATTTCTTCCATTTGTTCATTATCCATAGTG   480
Consensus         tcctaacaacccataaaaagtagtaattggttgcttttaaaccccaagctatttcttccatttgttcattatccatagtg

NbUGT17-cDNA.seq  GACATACTACCAAAAGCTACATAAACAACAGATCCTTCTGTTTTAGTATTTAGCCAGCTAATACATGTTGAAGCATCCAC   560
NbUGT17-DNA.seq   GACATACTACCAAAAGCTACATAAACAACAGATCCTTCTGTTTTAGTATTTAGCCAGCTAATACATGTTGAAGCATCCAC   560
Consensus         gacatactaccaaaagctacataaacaacagatccttctgttttagtatttagccagctaatacatgttgaagcatccac

NbUGT17-cDNA.seq  TTGGTATAGACTGAGACAATATTCAGTGTCATTTTCAACTCTATTGTCCAAATACAAAGATGGCAACGTTGGGCCTATTG   640
NbUGT17-DNA.seq   TTGGTATAGACTGAGACAATATTCAGTGTCATTTTCAACTCTATTGTCCAAATACAAAGATGGCAACGTTGGGCCTATTG   640
Consensus         ttggtatagactgagacaatattcagtgtcattttcaactctattgtccaaatacaaagatggcaacgttgggcctattg

NbUGT17-cDNA.seq  TCGACATTGGAATGACTTTTGACATTGCGTCTACT.............................................   675
NbUGT17-DNA.seq   TCGACATTGGAATGACTTTTGACATTGCGTCTACTACCTGCAATAAATATATTAATGAGAAATGGATTCAACTTATATAC   720
Consensus         tcgacattggaatgacttttgacattgcgtctact

NbUGT17-cDNA.seq  ................................................................................   675
NbUGT17-DNA.seq   AGTAAAAAGACAAATTTTTTACCTTATTAATGTAATTTAACCGGCTATGACATATCCTTAATGATATATTTAAATCATCA   800
Consensus

NbUGT17-cDNA.seq  ................................................................................   675
NbUGT17-DNA.seq   TGTAAAAGATAAGTAATAGTACTAGATACCAAATATTATTGCTTTTGATAACTTTATTATGTAATCATAAAATGTTTAAT   880
Consensus

NbUGT17-cDNA.seq  ................................................................................   675
NbUGT17-DNA.seq   TATTAGTTACTTTAAGTTTTATACTTTAATTAAATAAAAAAAAAGTTATTGAATACTTTGATACAAAACTGAACGTTTAC   960
Consensus

NbUGT17-cDNA.seq  ................................................................................   675
NbUGT17-DNA.seq   TATCCTTTTTTCCTGATTGGGTACTCAAAAGTACTTGACCAAACACAATTTGCTTATCAAATATTATAAAAAGAAGTCGT  1040
Consensus

NbUGT17-cDNA.seq  ................................................................................   675
NbUGT17-DNA.seq   CAAATGATTGACGAAACATAAATTACTACTCTCCAAAAAATATTTTTTCAAATATTACTTTTGAACAAAAACATTTATCA  1120
Consensus

NbUGT17-cDNA.seq  ................................................................................   675
NbUGT17-DNA.seq   AAATAAATAATTTTTGGCGGCGAAGTAGAGATCGCACCATTCAAGTGTAAGTATGAGAACGAGTGATAAGACATGAAATA  1200
Consensus

NbUGT17-cDNA.seq  ................................................................................   675
NbUGT17-DNA.seq   AAAATTCAAGTGAAAACAATTAAATACCTTAAATAGAAATAAAAAACCACCAAAGAATTTTAGTGGATGACGAATATTGT  1280
Consensus

NbUGT17-cDNA.seq  ........................ACCTCAGCCTCCAACTTGTAGAATGAGTTCACAAATACATAATCAGCTTTCTCCAC   731
NbUGT17-DNA.seq   CCACAAATAGTAAGTTTGAAACTAACCTCAGCCTCCAACTTGTAGAATGAGTTCACAAATACATAATCAGCTTTCTCCAC  1360
Consensus                                 acctcagcctccaacttgtagaatgagttcacaaatacataatcagctttctccac

NbUGT17-cDNA.seq  ATTTATAAACTGATTTAACACCAACTCAAAATACGCTGGATATGTACCATGGACATAAATGAATGACGGCATATCTCGAA   811
NbUGT17-DNA.seq   ATTTATAAACTGATTTAACACCAACTCAAAATACGCTGGATATGTACCATGGACATAAATGAATGACGGCATATCTCGAA  1440
Consensus         atttataaactgatttaacaccaactcaaaatacgctggatatgtaccatggacataaatgaatgacggcatatctcgaa

NbUGT17-cDNA.seq  GTTCGAGCTCCGGCAATCCCGGAATCCTCACCGGAGGTGAAGAAATTGGCAATGTCAATTTTCCGTGATGAACATAATAG   891
NbUGT17-DNA.seq   GTTCGAGCTCCGGCAATCCCGGAATCCTCACCGGAGGTGAAGAAATTGGCAATGTCAATTTTCCGTGATGAACATAATAG  1520
Consensus         gttcgagctccggcaatcccggaatcctcaccggaggtgaagaaattggcaatgtcaattttccgtgatgaacataatag

NbUGT17-cDNA.seq  TAAATATAGTTCACAGAACATGCTTGAGTGAAAAAACAAGATCCAATAAGGCCATGATCTTTGGCTACGTCTAAAGCCCA   971
NbUGT17-DNA.seq   TAAATATAGTTCACAGAACATGCTTGAGTGAAAAAACAAGATCCAATAAGGCCATGATCTTTGGCTACGTCTAAAGCCCA  1600
Consensus         taaatatagttcacagaacatgcttgagtgaaaaaacaagatccaataaggccatgatctttggctacgtctaaagccca

NbUGT17-cDNA.seq  TGGCATGAAAGCATCATAAATAACACAAGTAATAGGAAACTCTGATTTTTCGTATTTCTTGATAAGGTCCTCTAGGGTTT  1051
NbUGT17-DNA.seq   TGGCATGAAAGCATCATAAATAACACAAGTAATAGGAAACTCTGATTTTTCGTATTTCTTGATAAGGTCCTCTAGGGTTT  1680
Consensus         tggcatgaaagcatcataaataacacaagtaataggaaactctgatttttcgtatttcttgataaggtcctctagggttt

NbUGT17-cDNA.seq  GCGAGCCGATTTTCTTGAAGCGTTCGAGATAGGTGACTATGCTGTCAGCTTCAGCGTAGCCACCTTTGTCGAACCCATCG  1131
NbUGT17-DNA.seq   GCGAGCCGATTTTCTTGAAGCGTTCGAGATAGGTGACTATGCTGTCAGCTTCAGCGTAGCCACCTTTGTCGAACCCATCG  1760
Consensus         gcgagccgattttcttgaagcgttcgagataggtgactatgctgtcagcttcagcgtagccacctttgtcgaacccatcg

NbUGT17-cDNA.seq  GAAATGGTATCGATACTAACGTTAATGGAAATTGGACGGACTGAATGGGAAATGAAGTTAGTAATGGCTAAAGTGGTCTT  1211
NbUGT17-DNA.seq   GAAATGGTATCGATACTAACGTTAATGGAAATTGGACGGACTGAATGGGAAATGAAGTTAGTAATGGCTAAAGTGGTCTT  1840
Consensus         gaaatggtatcgatactaacgttaatggaaattggacggactgaatgggaaatgaagttagtaatggctaaagtggtctt

NbUGT17-cDNA.seq  TACACTTTTGGAAACTAAACGTTTGCAGAATTGAAGCATAGGGTTTAAGTGGCCTTGGCTTGGATAAGGAAGAGCTAAAA  1291
NbUGT17-DNA.seq   TACACTTTTGGAAACTAAACGTTTGCAGAATTGAAGCATAGGGTTTAAGTGGCCTTGGCTTGGATAAGGAAGAGCTAAAA  1920
Consensus         tacacttttggaaactaaacgtttgcagaattgaagcatagggtttaagtggccttggcttggataaggaagagctaaaa

NbUGT17-cDNA.seq  TATGAGCTACATACTTTTTGTTATTCAACAAGTCCATCTTTCTCCTTTTGCTTTATTGGGAGTCTAATTTTTCCTTCCTC  1371
NbUGT17-DNA.seq   TATGAGCTACATACTTTTTGTTATTCAACAAGTCCATCTTTCTCCTTTTGCTTTATTGGGAGTCTAATTTTTCCTTCCTC  2000
Consensus         tatgagctacatactttttgttattcaacaagtccatctttctccttttgctttattgggagtctaatttttccttcctc

NbUGT17-cDNA.seq  TATCACTTGTGAGAGGA                                                                1388
NbUGT17-DNA.seq   TATCACTTGTGAGAGGA                                                                2017
Consensus         tatcacttgtgagagga
```

**Figure S2.** Detection of the presence of introns for *NbUGT12*, *NbUGT16* and *NbUGT17*. Matching the full-length nucleotide sequences of *NbUGT12*, *NbUGT16* and *NbUGT17* cloned from cDNA and DNA respectively.
