# Supplementary material for: Genome-Wide Analysis and Identification of UDP Glycosyltransferases Responsive to Chinese Wheat Mosaic Virus Resistance in Nicotiana benthamiana
Source: Viruses. 2024 Mar 22;16(4):489. doi: 10.3390/v16040489 (PMC11054786; doi:10.3390/v16040489)
Supplement: Supplementary file 1 [file viruses-16-00489-s001.zip › viruses-2868224-supplementary/Supplementary File-viruses-2868224/Table S1.pdf]

Table S1. Amino acid sequences of ten conserved motifs

Motif	Width	Sequence
Motif-1	41	GWAPQLEILAHPSIGGFLTHCGWNSTLESJSFGVPMIAWPM
Motif-2	30	QPPKSVVYVSFGSEASLSKEQLKEJALGLE
Motif-3	29	KLHVVMVPFPAQGHJNPLLELAKLLASRG
Motif-4	11	SGQPFJWVLRD
Motif-5	18	TLDVAKELNIPRIVFFTS
Motif-6	11	GFEERTKGRGJ
Motif-7	11	FADQPTNAKLV
Motif-8	21	RKRAKELKELAKKAVEEGGSS
Motif-9	21	GIVTREEIEKAVRELMESEEG
Motif-10	15	DGIJVNTFEELEGEY
