# Supplementary material for: Genome-Wide Analysis and Identification of UDP Glycosyltransferases Responsive to Chinese Wheat Mosaic Virus Resistance in Nicotiana benthamiana
Source: Viruses. 2024 Mar 22;16(4):489. doi: 10.3390/v16040489 (PMC11054786; doi:10.3390/v16040489)
Supplement: Supplementary file 1 [file viruses-16-00489-s001.zip › viruses-2868224-supplementary/Supplementary File-viruses-2868224/Table S2.pdf]

Table S2. Nucleotide sequences of silent fragments

pTRV2-NbUGT12	GTTGGCTCCGATACTCTGACTCAACTTATTAAGAAATTAGAAAGT<br>TGTGAGTACCCTGTGAATTGCATAGTTTATGATCCATTCCTTCCTT<br>GGGCTGTTGAAGTAGCGAAGGATCTTGGATTAGTTAATGCTGCTT<br>TTTTCACACAAAATTGTGTAGTAGATAACATTTACTACCATGTACA<br>TAAAGGGGTACTAAAACTTCCTCCTACTCAAGTTGATGGACAAA<br>TATTAATTCCTGGATTATCAAGTACAATTGAGAGTTCAGATGTAC<br>CTAGTTTTGAGTCTAGTCCTCAATCAGAT
pTRV2-NbUGT16	ACAAGTACTAATATTATCACATGCTTCTGTCGGGGGATTCGTGAC<br>TCATTGTGGATGGAATTCGAGTATAGAAGGAATATCAACTGGCGT<br>GCCAATGATCACTTGGCCATTATTTGCTGAGCAATTTTGTAATGA<br>GAGGCTTATTACGAATGTTCTCAAGACAGGAGTAAAGTCTGGCG<br>TCGAGAATCCTGTTATGTTTTTAGAGGAGGAAAAAGTGGATACTC<br>AAGTGAACAAAGATGACATTAAAATGGTTATTGAAAAATTAATG<br>GGTGAAGAAGAGGAAGCAAAAATAAGAAGAGA
pTRV2-NbUGT17	TGGACTTGTTGAATAACAAAAAGTATGTAGCTCATATTTTAGCTC<br>TTCCTTATCCAAGCCAAGGCCACATAAACCCTATGCTTCAATTCT<br>GCAAACGTTTAGTTTCCAAAAGTGTAAAGACCACTTTAGCCATT<br>ACTAACTTCATTTCCCATTCAGTCCGTCCAATTTCCATTAACGTTA<br>GTATCGATACCATTTCCGATGGGTTCGACAAAGGTGGCTACGCTG<br>AAGCTGACAGCATAGTCACCTATCTCGAACGCTTCAAGAAAATC<br>GGCTCGCAAACCCTAGAGGACCTTATCAAG
