# Supplementary material for: Genome-Wide Analysis and Identification of UDP Glycosyltransferases Responsive to Chinese Wheat Mosaic Virus Resistance in Nicotiana benthamiana
Source: Viruses. 2024 Mar 22;16(4):489. doi: 10.3390/v16040489 (PMC11054786; doi:10.3390/v16040489)
Supplement: Supplementary file 1 [file viruses-16-00489-s001.zip › viruses-2868224-supplementary/Supplementary File-viruses-2868224/Table S3.pdf]

Table S3. The primers sequences used in this study

Prime Name	Prime Sequence(5' → 3')
NbUGT1-RT-F	CGATGGTCACAAATCAAACG
NbUGT1-RT-R	ACATAAGCCAAGAAAAGCGG
NbUGT2-RT-F	GCTGAAGGATTGGGCACTCA
NbUGT2-RT-R	CATCTCCTTGCGATTGCCAC
NbUGT3-RT-F	CGTTGCTGGAAAAGGAAGAT
NbUGT3-RT-R	GCGGTTTGGTTAGACTTGAG
NbUGT6-RT-F	CTTCTTCCTCCAGTCTACCC
NbUGT6-RT-R	GAATGTTTGCTGGCTGTTTG
NbUGT8-RT-F	AACTAAAAGACTTCAACCCC
NbUGT8-RT-R	AACAGAGTCTGATAAAGAG
NbUGT9-RT-F	ATTGGATACCTGGCATGGAG
NbUGT9-RT-R	AAGTGTGAAAGGGTCCAAGA
NbUGT10-RT-F	GATGTTCTGTCCCGATTTGG
NbUGT10-RT-R	AGTTGATTTCCTCGGCTACA
NbUGT11-RT-F	TACTTCAATGGTATCCGTCGT
NbUGT11-RT-R	TTCCCAATGTTCCAAAGCAC
NbUGT12-RT-F	CCGATGGCAAAAGAGTGCTT
NbUGT12-RT-R	TTGGGGACACCATGAAACCA
NbUGT13-RT-F	TTGAAGGCAGGCAAATCGGA
NbUGT13-RT-R	CATGGGCTTTCCCTCACAGA
NbUGT15-RT-F	TGCATTCTGATCAGCCGAGG
NbUGT15-RT-R	CCCACCATCCATCACTGACA
NbUGT16-RT-F	GAACTCGGACTTGCATTAGAG
NbUGT16-RT-R	TCCATCCACAATGAGTCACG
NbUGT17-RT-F	TCCAGCGTATTTTGAGTTGG
NbUGT17-RT-R	GCATCCACTTGGTATAGACTG
NbUGT18-RT-F	GCTTGCATGGGGTTTAGAAT
NbUGT18-RT-R	TGTGTCAAAAATCCACCCAC
NbUGT19-RT-F	TGCAAGAACCACTGGAACAG
NbUGT19-RT-R	TGCTCAAACGGAGACACCTG
NbUGT21-RT-F	AAAGGCACGAAAGCCCGATA
NbUGT21-RT-R	GGAGGCATCTCCTCTTCAGG
NbUGT22-RT-F	ACAATCAGCCACCGAACTCT
NbUGT22-RT-R	GAGTCCAAAACCCGCCTACA
NbUGT23-RT-F	TTGGCCCACTCTGCTATTGG
NbUGT23-RT-R	ACGTGTCCTTCATGTCCACC
NbUGT24-RT-F	AAACTCGCGGACAAGGGATT
NbUGT24-RT-R	AAGGGCGTGATTGAGATGGGG
NbUGT25-RT-F	TTCGAGATGCACAAGTGGCT
NbUGT25-RT-R	CCGTGAGAGGGTCAAAACCA
NbUGT26-RT-F	TGTTGAAAATGGCTTGCCCG
NbUGT26-RT-R	CTGATGCTCTCCCCAACACA

CWMV CP-F	ACGAGGACGAGTGTGTTGTC
CWMV CP-R	CACCTTTAGCAGCACCGAGA
Nbactin-F	AGGCTGTTCTTTCCCTCTATGC
Nbactin-R	CAACTTCTCCTTCACATCCCTAAC
NbUGT12-A-F	GTCATCCCATCAATTGACAAG
NbUGT12-A-R	TCTTTGTGTAGGCTCAATTTCC
NbUGT16-A-F	AGGCACGCAGCCGAACATTTTC
NbUGT16-A-R	ATGGATCACTAACAGCTTGC
NbUGT17-A-F	TCCTCTCACAAGTGATAGAGG
NbUGT17-A-R	TGCAGTGATTAAATCACTGATC
505-NbUGT12-F	GGGGACAAGTTTGTACAAAAAAGCAGGCTTCATGAGTACTACTCACAAAGCTC
505-NbUGT12-R	GGGGACCACTTTGTACAAGAAAGCTGGGTCGGAAATAGTAATCAAACTGG
505-NbUGT16-F	GGGGACAAGTTTGTACAAAAAAGCAGGCTTCATGGCAGTTCTTACTAATGAGC
505-NbUGT16-R	GGGGACCACTTTGTACAAGAAAGCTGGGTCGACATCAAATTGATTTTCC
505-NbUGT17-F	GGGGACAAGTTTGTACAAAAAAGCAGGCTTCATGGACTTGTTGAATAAC
505-NbUGT17-R	GGGGACCACTTTGTACAAGAAAGCTGGGTCTTTAGTACATTTTGATAC
pTRV2-NbUGT12V-F	CGCGGATCCGTTGGCTCCGATACTCTGACTC
pTRV2-NbUGT12V-R	TCCCCCGGGATCTGATTGAGGACTAGACTC
pTRV2-NbUGT16V-F	CGCGGATCCACAAGTACTAATATTATCACATGC
pTRV2-NbUGT16V-R	TCCCCCGGGTCTCTTCTTATTTTTGCTTCC
pTRV2-NbUGT17V-F	CGCGGATCCTGGACTTGTTGAATAAC
pTRV2-NbUGT17V-R	TCCCCCGGGCTTGATAAGGTCCTCTAGG
pTRV-RT-NbUGT12-F	TGTCATCCCATCAATTGACAAG
pTRV-RT-NbUGT12-R	ATGGCCTTGATTGACACTGAAG
pTRV-RT-NbUGT16-F	ACTAAATTCAACCAAATGGCAG
pTRV-RT-NbUGT16-R	TAGCCCTGCTTCTGAGCTTG
pTRV-RT-NbUGT17-F	AGGATTTGGCTAAGGAAGCTG
pTRV-RT-NbUGT17-R	TGGTTTCACTTGTTCAGTAGC
