# Supplementary material for: Genome-Wide Analysis and Identification of UDP Glycosyltransferases Responsive to Chinese Wheat Mosaic Virus Resistance in Nicotiana benthamiana
Source: Viruses. 2024 Mar 22;16(4):489. doi: 10.3390/v16040489 (PMC11054786; doi:10.3390/v16040489)
Supplement: Supplementary file 1 [file viruses-16-00489-s001.zip › viruses-2868224-supplementary/Supplementary File-viruses-2868224/Table S4.pdf]

Table S4. The corresponding genes' names

Name	GENE ID	ACCESSION	IN	SL	GROUP
NbUGT1	Niben101Scf00270g15011.1	MT945324.1	1	PlasmaMembrane	E
NbUGT2	Niben101Scf00492g00010.1/Niben101Scf07089g00006.1	MT945394.1	0/0	Cytoplasmic/Cytoplasmic Mitochondrial	O
NbUGT3	Niben101Scf01017g03006.1	MT945405.1	1	Cytoplasmic	L
NbUGT5	Niben101Scf01834g04027.1/Niben101Scf01999g03009.1/Niben101Scf17612g02009.1	MT945400.1	3/0/0	Cytoplasmic/Cytoplasmic/Cytoplasmic	A
NbUGT6	Niben101Scf01980g10004.1	MT945328.1	1	Cytoplasmic	G
NbUGT8	Niben101Scf02405g04013.1	MT945368.1	3	Cytoplasmic	D
NbUGT9	Niben101Scf02537g09004.1	MT945331.1	1	Cytoplasmic	G
NbUGT10	Niben101Scf02653g06007.1	MT945329.1	1	PlasmaMembrane Cytoplasmic	H
NbUGT11	Niben101Scf02807g03003.1	MT945343.1	2	Cytoplasmic Chloroplast	E
NbUGT12	Niben101Scf03012g03021.1	MT945323.1	1	Cytoplasmic	L
NbUGT13	Niben101Scf03536g01017.1	MT945387.1	1	Cytoplasmic	H
NbUGT15	Niben101Scf03973g00012.1/Niben101Scf03709g03002.1	MT945375.1	5/1	Mitochondrial Cytoplasmic/Cytoplasmic	O
NbUGT16	Niben101Scf04875g02008.1	MT945336.1	1	Cytoplasmic	D
NbUGT17	Niben101Scf05415g00003.1	MT945322.1	1	PlasmaMembrane Cytoplasmic	L
NbUGT18	Niben101Scf06112g01008.1	MT945401.1	1	Cytoplasmic	E
NbUGT19	Niben101Scf06344g00005.1	MT945327.1	0	Cytoplasmic	P
NbUGT21	Niben101Scf08015g03004.1	MT945339.1	0	Cytoplasmic PlasmaMembrane	A
NbUGT22	Niben101Scf08467g03008.1	MT945349.1	1	Cytoplasmic Nuclear	H
NbUGT23	Niben101Scf08549g02008.1	MT945377.1	1	PlasmaMembrane	H
NbUGT24	Niben101Scf09225g04002.1/Niben101Scf01763g00016.1	MT945325.1	1/0	Cytoplasmic/Cytoplasmic	A
NbUGT25	Niben101Scf11008g01002.1	MT945346.1	0	Cytoplasmic	O
NbUGT26	Niben101Scf12919g00023.1	MT945371.1	0	Cytoplasmic	P

IN, intron number; SL, subcellular location.
